# Enhanced single-isomer separation and pseudoenantiomer resolution of new primary rim heterobifunctionalized α-cyclodextrin derivatives

**DOI:** 10.3762/bjoc.14.261

**Published:** 2018-11-13

**Authors:** Iveta Tichá, Gábor Benkovics, Milo Malanga, Jindřich Jindřich

**Affiliations:** 1 Department of Organic Chemistry, Faculty of Science, Charles University, Hlavova 8, 128 43 Prague 2, Czech Republic; 2 CycloLab, Cyclodextrin R&D Ltd., Illatos út 7, H-1097 Budapest, Hungary

**Keywords:** diazido-alpha-cyclodextrin, heterobifunctionalized alpha-cyclodextrin, homobifunctionalized alpha-cyclodextrin, regioisomers, regioselectivity

## Abstract

The synthesis of batch-to-batch reproducible cyclodextrin (CD) derivatives often requires functionalization at specific positions of the CD skeleton. However, the regioselective preparation of this type of CD derivatives remains a challenge in synthetic chemistry. Thus, the present study aimed to prepare all positional regioisomers on the primary rim of homobifunctionalized diazido-α-CDs by selective substitution on the primary rim. Specifically, three positional regioisomers 6^A^,6^B^-, 6^A^,6^C^-, and 6^A^,6^D^-diazido-α-CDs were prepared via different intermediates (using sulfonylation with capping agents, bromination and tosylation). Furthermore, heterobifunctionalized 6^A^-azido-6^X^-mesitylenesulfonyl-α-CDs were also synthesized, and all regioisomers were successfully separated by HPLC. Moreover, the heterobifunctionalized α-CD regioisomers were isolated in gram-scale quantities, isomers AB and AC in the form of a pseudoenantiomeric mixture. The pseudoenantiomers AC/CA and AB/BA were resolved on an analytical scale by HPLC–MS at 10 °C. Thus, the presented synthetic and analytical methods for homo- and heterodisubstituted α-CDs are efficient and reproducible for the preparation of various pure regioisomeric CD derivatives. Accordingly, our findings indicate, (i) the versatility of selectively modified azido and mesitylene CD skeletons in preparing new types of α-CD derivatives and (ii) the potential of using resolved α-CD pseudoenantiomers in other research fields such as organocatalysis.

## Introduction

Cyclodextrins, cyclic cone-shaped oligosaccharides [1], have long attracted interest for their properties in host–guest complexes both in research and in pharmaceutical and food industry, among other fields [2]. Recent studies have improved the properties of CD scaffolds primarily by selective persubstitution and monosubstitution [2–3], thereby broadening their application. However, the synthesis of selectively disubstituted CD derivatives with the same or different functional groups at selected positions remains a challenging task because of the similar reactivity of the hydroxy groups.

**Figure 1:**
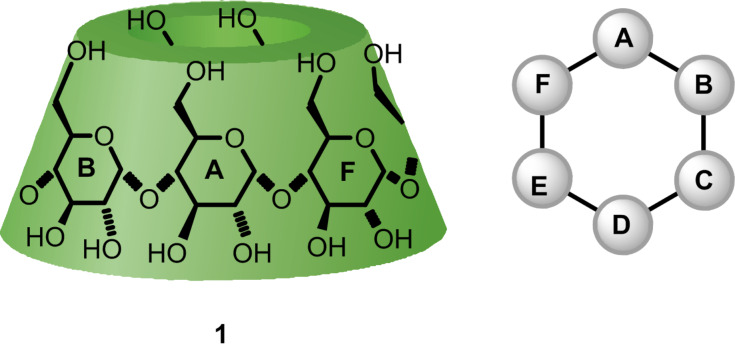
Schematic representation of native α-CD (**1**) and top view of its primary rim with alphabetic clockwise numbering of the glucose units.

Nevertheless, selectively disubstituted CD derivatives are used to prepare artificial enzymes [4–5], metallocatalysts and organocatalysts [6–8] or amino acid mimics [9]. Among the methods used to prepare these derivatives on α-, β- or γ-CDs, disubstitutions of the α-CD primary rim occurring at two different glucose units (Figure 1) stands out for well-explored direct and indirect approaches.

The indirect preparation of selectively disubstituted CDs, including protecting and deprotecting steps, was introduced by Sinaÿ et al. [10] and continued in the studies by Sollogoub et al. [11–12]. The addition of diisobutylaluminum hydride (DIBAL-H) to perbenzylated CDs exclusively formed a single AD regioisomer on the primary rim of α-CD.

Conversely, the direct, straightforward modification is mostly performed using capping agents. For example, Tabushi et al. [13–15] studied regioselectively capped-β-CDs with disulfonyl chlorides. Subsequently, Fujita et al. [16] introduced the disulfonyl groups on the primary rim of α-CDs, albeit using non-capping reagents, mesitylenesulfonyl chloride and observed almost no preferential selectivity for AB, AC or AD regioisomers. Breslow et al. [17] used the 1,3-dimethoxy-4,6-benzenedisulfonyl chloride as a capping agent to prepare the AB regioisomer. Subsequently, Fujita et al. [18] selectively capped the primary rim of α-CD using a special, sterically hindered sulfonylation agent, dibenzofuran-2,8-disulfonyl chloride, but observed only a negligible selectivity towards the AC regioisomer. Notwithstanding, these researchers were more successful in achieving selectivity for the AB regioisomer by introducing auxiliary groups (phthalimide) [19]. Armspach et al. investigated the regioselective synthesis of tris(4-*tert*-butylphenyl)methyl ethers on the primary rim of α-CD which resulted in predominant AD species [20]. Trityl groups for regioselective bi- and trifunctionalization of α-CD were also investigated [21]. Thus, the use of either a direct single-step or indirect method generally led to selectively homobifunctionalized CDs.

In contrast, heterobifunctionalized CDs have been prepared via multistep synthesis (depending on the steric hindrance of the moiety on the CD scaffold and on the bulkiness of reagents [22]) or by modification of homobifunctionalized CDs [23–24]. These and previous studies [25–26] confirmed that CD derivatives with bulky substituents such as tosyl groups can be separated by reversed-phase column chromatography and HPLC separation methods can be easily scaled up to obtain grams of pure regioisomers. However, to the best of our knowledge, no such comparative regiochemical study of homobifunctionalized α-CDs prepared from the most commonly used CD intermediates has been conducted thus far although the regioisomer pattern of these intermediates is crucial for the investigation of new single isomer CDs with reproducible and definite structures. Furthermore, the direct synthesis of new pure heterobifunctionalized α-CD derivatives remains a challenging task due to problematic regioisomer separations. Subsequently, the phenomenon of pseudoenantiomers on the heterobifunctionalized α-CD primary rim (AB/BA, AC/CA, AD/DA) has not yet been studied in detail by HPLC–MS methods. Nevertheless, the studies on the perbenzylated α-CD skeletons by Sollogoub et al. [27] did not confirm an existence of an AD/DA pseudoenantiomeric pair.

The present study reports a fast and reproducible approach for the synthesis of six types of 6^A^,6^X^-disubstituted α-CD intermediates. Initially, these intermediates were transformed into stable, non-hydrolyzing 6^A^,6^X^-diazido-6^A^,6^X^-dideoxy-α-CDs (6^A^,6^X^-diazido-α-CDs). The regioisomeric pattern of each 6^A^,6^X^-diazido-α-CD was thoroughly assessed, compared and used to develop methods for the separation of new regioselectively pure α-CD derivatives with two non-identical functional groups, such as azido and mesitylenesulfonyl. This comparison between regioisomeric patterns of disubstituted α-CD derivatives is the key to the development of new heterodisubstituted α-CDs with exact structures.

Thus in this study, we prepared all possible regioisomers of primary rim-disubstituted α-CD derivatives using the most common CD intermediates by commonly used derivatization (bromination, tosylation and capping). The three positional isomers (AB, AC, AD) were separated by ad hoc-developed HPLC methods, and the regioisomeric pattern was elucidated. Furthermore, new heterobifunctionalized α-CD derivatives with easily transformable functional groups were also prepared and unambiguously confirmed, to study in detail possible α-CD primary rim pseudoenantiomers. These pseudoenantiomers were undoubtedly confirmed via HPLC–MS methods. Moreover, these regioselectively pure derivatives will be further transformed to create key intermediates for a wide range of new single-isomer CD derivatives, thereby opening a new area of research in CD chemistry.

## Results and Discussion

### Synthesis of homobifunctionalized α-CD derivatives

The first part of this study focused on the synthesis of all primary rim regioisomers of 6^A^,6^X^-diazido-α-CDs through different types of reactions (two different capping agents; direct and indirect brominations; tosylations in pyridine), their analytical separation and regioisomer elucidation using ad hoc-developed HPLC methods.

The synthetic approach started with the preparation of 6^A^,6^X^-capped α-CD derivatives. From special capping agents previously used on α-CD [17–18,26], we selected the most common, relatively inexpensive and commercially available *m*-benzenedisulfonyl chloride and biphenyl-4,4´-disulfonyl chloride. The prepared 6^A^,6^X^-disulfonyl-capped CDs **2** and **3** were immediately converted to the more stable 6^A^,6^X^-diazido-α-CDs **4** to prevent hydrolysis. However, the yields were low because the hydrolysis of sulfonyl groups could not be completely suppressed. The prepared 6^A^,6^X^-diazido-α-CDs were then separated by HPLC to afford the single AB, AC and AD regioisomers.

**Scheme 1:**
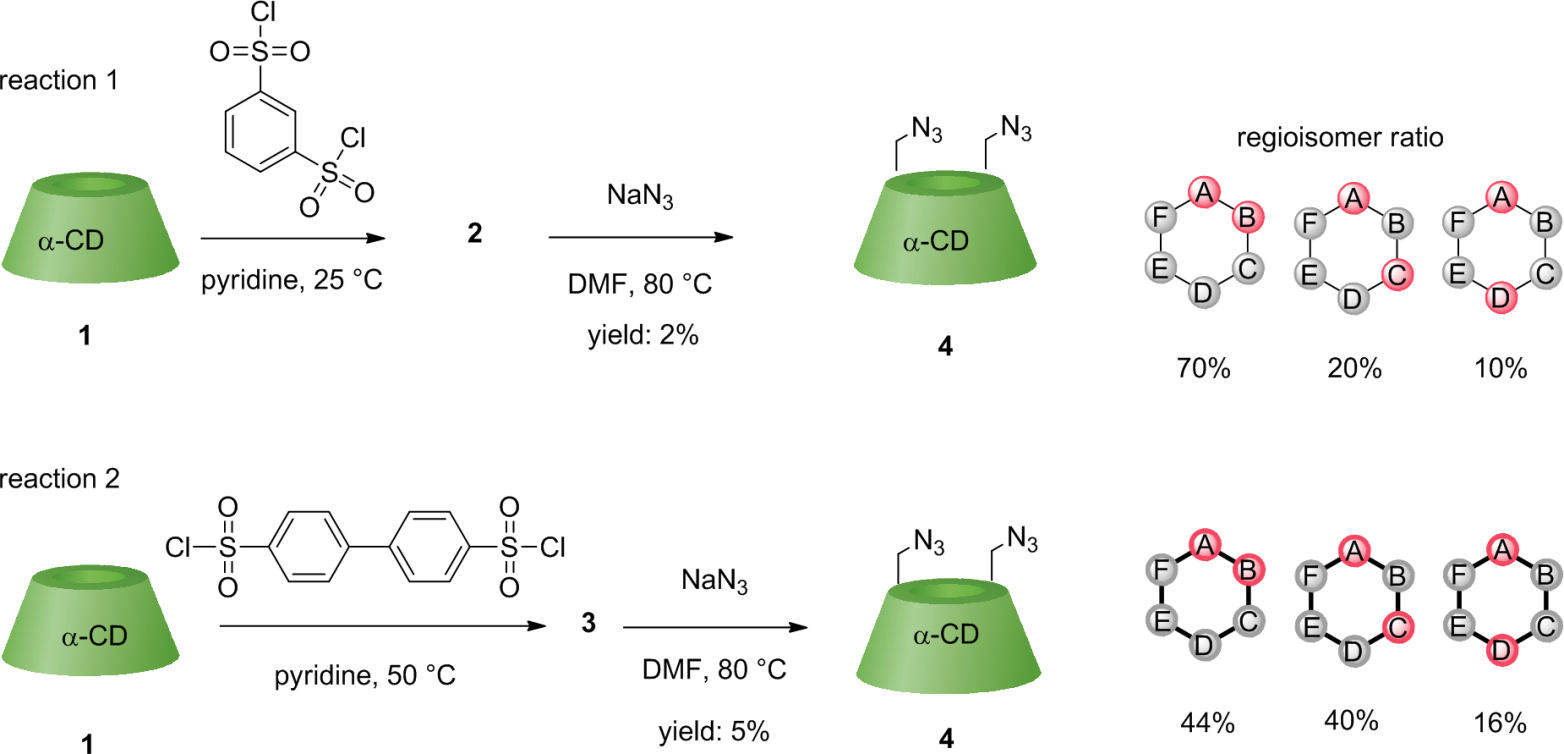
Synthesis of 6^A^,6^X^-diazido-α-CD derivatives **4** via 6^A^,6^X^-capped α-CDs **2** and **3** and their regioisomeric ratios.

After the reaction with *m*-benzenedisulfonyl chloride (reaction 1, Scheme 1), a ratio AB/AC/AD = 70:20:10 was found, thus showing that the AB regioisomer is the predominant species, in agreement with previous findings [17]. This AB regioisomer was used as one standard in subsequent HPLC analyses. The regioisomeric ratios are given as the percentages of areas of the HPLC peaks (100 correspond to the sum of areas of AB, AC and AD peaks). Conversely, using of biphenyl-4,4´-disulfonyl chloride (reaction 2, Scheme 1), the ratio AB/AC/AD = 44:40:16 indicated almost no selectivity. This is in contrast to Tabushi [14] who observed 84% selectivity for the AD regioisomer on β-CD. This different regioisomeric ratio could be caused by the formation of intermolecular bridges among α-CDs during the capping which resulted in the higher ratio for AC and AD regioisomers. Nevertheless, the fast hydrolysis of these intermediates did not allow to study this phenomenon in more detail.

**Scheme 2:**
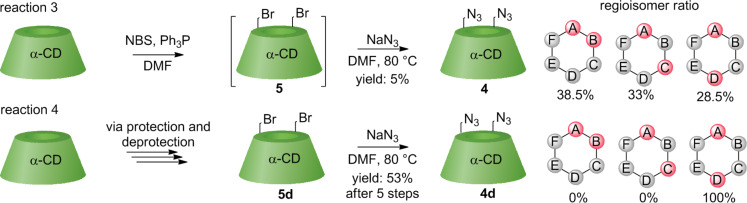
Synthesis of 6^A^,6^X^- and 6^A^,6^D^-diazido-α-CDs via 6^A^,6^X^-dibromo-α-CD **5**, 6^A^,6^D^-dibromo-α-CD **5d** intermediates and their regioisomeric ratios.

The second synthetic approach for homobifunctionalized α-CDs was based on the preparation of 6^A^,6^X^-dibromo-α-CD intermediates, both directly (**5**) and indirectly (**5d**). On one hand, a direct bromination of α-CD was performed under Vilsmeier/Haack conditions (reaction 3, Scheme 2) with *N*-bromosuccinimide (NBS) and Ph_3_P in *N,N-*dimethylformamide (DMF). Previous studies have shown that this reaction is highly regioselective for the primary rim of CD [28]. After a fast work-up, the reaction mixture, consisting of unreacted α-CD, mono-, di- and trisubstituted products, was converted into the corresponding 6^A^,6^X^-diazido-α-CDs **4** [29]. The ratio of the regioisomers was assessed as AB/AC/AD = 39:33:29. The distribution of the positional isomers is almost statistical, and practically no regioselectivity was observed.

On the other hand, the dibromo-α-CD derivative was also prepared using a four-step indirect method involving DIBAL-H deprotection (reaction 4, Scheme 2) [10,30]. This method generated the AD regioisomer exclusively without AB or AC regioisomers as byproducts (based on the published mechanism [10]). Nevertheless, it is a method demanding much more time and reagents compared to the direct dibromination. This finding was the second key data for the unambiguous elucidation of the regioisomer pattern, and the AD regioisomer was used as the second standard (the AB reference comes from reaction 1). The 6^A^,6^D^-dibromo-α-CD intermediate **5d** was converted again to 6^A^,6^D^-diazido-α-CD **4d**. The regioisomer ratio AB/AC/AD = 0:0:100 observed thus undoubtedly confirmed the selectivity for the AD regioisomer.

**Scheme 3:**
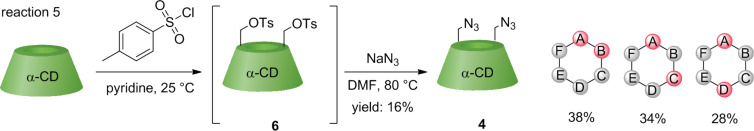
Synthesis of 6^A^,6^X^-diazido-α-CDs via 6^A^,6^X^-ditosyl-α-CD intermediates **6** and their regioisomeric ratios.

The last synthetic approach for the homobifunctionalized α-CDs included the preparation of 6^A^,6^X^-ditosyl-α-CD **6** under classical tosylation conditions with an excess of tosyl chloride (TsCl) in pyridine (reaction 5, Scheme 3). A previous study has shown [31] that tosyl groups exclusively attach to the primary rim of α-CD. Subsequently, the reaction mixture was converted into 6^A^,6^X^-diazido-α-CD **4** and the HPLC method revealed a regioisomeric ratio AB/AC/AD = 38:34:28. Therefore, the α-CD ditosylation is still a primary rim-selective process with negligible selectivity towards the AB regioisomer.

The regioisomeric ratios from reactions 1–5 provided crucial data for the unambiguous determination of the regioisomeric pattern of primary rim-homodisubstituted α-CDs, and the comparative analysis revealed the regioisomeric ratios of the most commonly used disubstituted CD intermediates.

### HPLC Analysis of homobifunctionalized α-CDs

**Figure 2:**
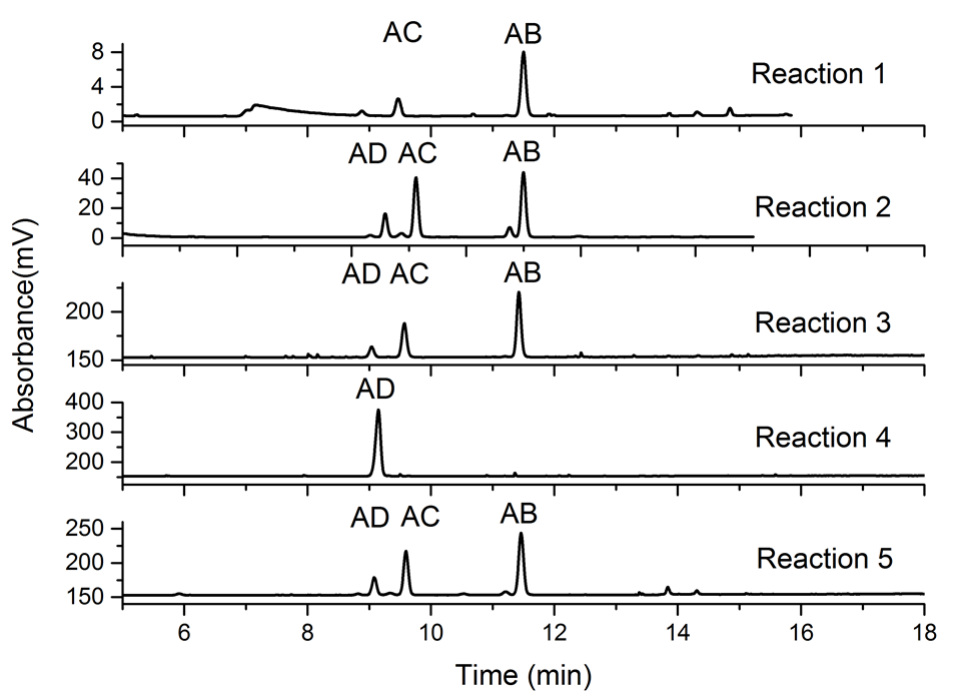
HPLC chromatograms of 6^A^,6^X^-diazido-α-CDs **4** of the reactions 1–5, with ACN/water gradient elution and ELS detection.

The regioisomeric ratios of the series of 6^A^,6^X^-diazido-α-CDs were determined using ad hoc-developed HPLC methods with an evaporative light scattering (ELS) detector and acetonitrile (ACN)/water gradient elution. The retention times of each 6^A^,6^X^-diazido-α-CD **4** from reactions 1 to 5 were compared (Figure 2). Nevertheless, the crucial information for the determination of the regioisomeric pattern was gathered in reactions 1 and 4. Reaction 1 yielded mostly AB regioisomer **4b**, whereas reaction 4 exclusively generated pure AD regioisomer **4d**. These compounds, previously prepared by disulfonyl capping [17] (reaction 1) and DIBAL-H deprotection [10] (reaction 4) were used as standards. The regioisomeric pattern was thus concluded: the AD regioisomer eluted first, the AC regioisomer second and the AB regioisomer last.

### Synthesis and large-scale separation of heterobifunctionalized α-CDs

After elucidating the homofunctionalized regioisomeric pattern, we prepared the target 6^A^,6^X^-heterobifunctionalized α-CDs. Mesitylenesulfonyl was chosen as the appropriate functional group for insertion in the α-CD skeleton because it is sufficiently stable for reverse column chromatography and more sterically hindered than a tosyl group. The idea of attaching the mesitylenesulfonyl moiety instead of the tosyl group to the β-CD skeleton was first reported in the study by Tang et al. [32] who observed regioselectivity for the AC isomer. In turn, the azido functional group was selected for its stability and versatility of subsequent modification: this group can be reduced to an amino group able to react with a large variety of electrophiles and is commonly used in click reactions [33].

**Scheme 4:**
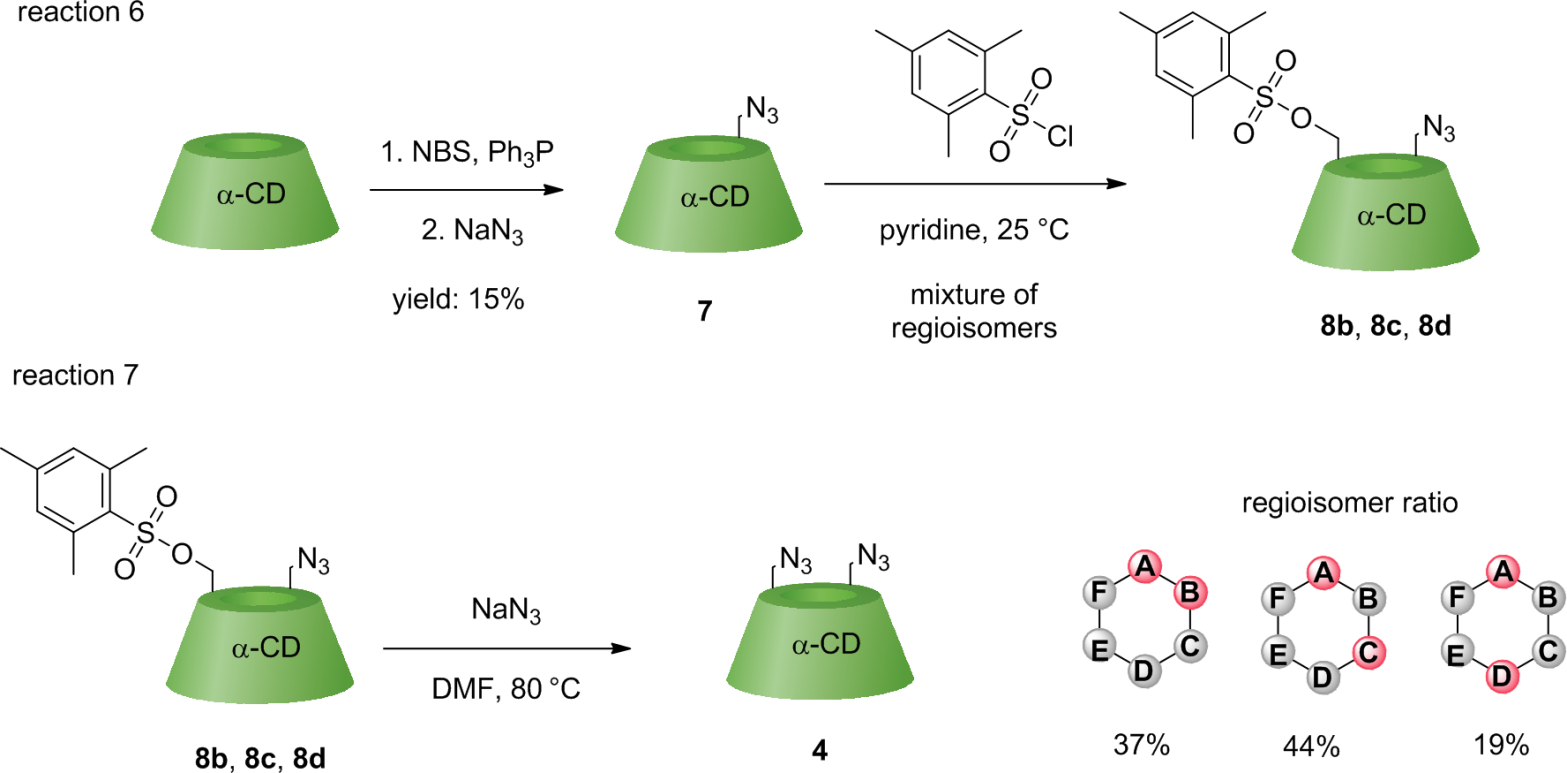
Synthesis of 6^A^-azido-6^X^-mesitylenesulfonyl-α-CD **8** and conversion into 6^A^,6^X^-diazido-α-CD **4**.

At first, 6^A^-azido-α-CD **7** was prepared via bromination of α-CD (as previously described for direct bromination) [28]. Then, 6^A^-azido-6^X^-mesitylenesulfonyl-α-CD **8** was prepared (reaction 6, Scheme 4) by adapting the reported procedure for β-CD based on mesitylenesulfonyl chloride in pyridine [32]. After work-up of the reaction, a small part of the crude mixture was transformed into the corresponding 6^A^,6^X^-diazido-α-CD **4** (reaction 7, Scheme 4), and HPLC retention times were compared with data from reactions 1–5 to elucidate the regioisomeric pattern.

**Figure 3:**
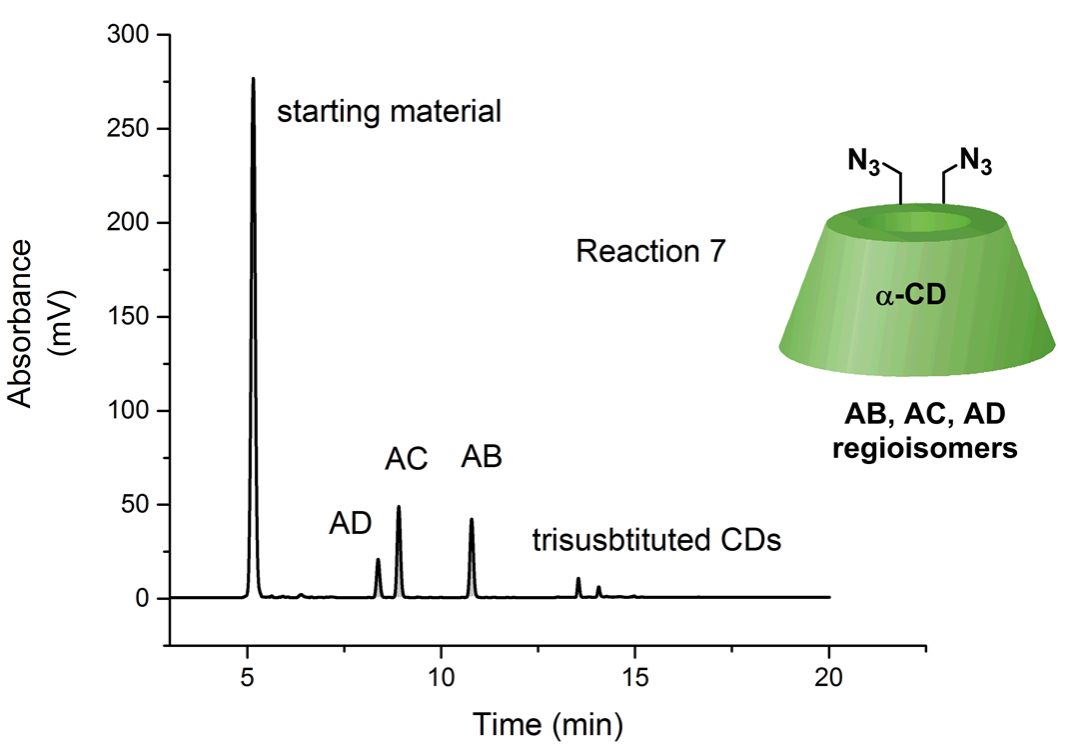
HPLC chromatograms of reaction 7 with separated 6^A^-azido-α-CD **7** as starting material and regioisomers of 6^A^,6^X^-diazido-α-CD **4**.

The analytical HPLC analysis of 6^A^,6^X^-diazido-α-CD **4** (prepared in reaction 7) revealed the regioisomeric ratio AB/AC/AD = 37:44:19 (Figure 3). This regioisomeric ratio of AB and AC was similar, however, the AD derivative was slightly suppressed. The measurements were performed with the crude reaction mixture comprising the starting material **7**, all regioisomers of 6^A^,6^X^-diazido-α-CD **4** and oversubstituted 6^A^,6^X^,6^Y^-triazido-α-CD derivatives. The HPLC signals of all components of the reaction mixture were nicely separated and thus allowed the isolation of each regioisomer in a single step with no need to separate the starting material first and then separate the corresponding 6^A^,6^X^-diazido-α-CD regioisomers.

**Figure 4:**
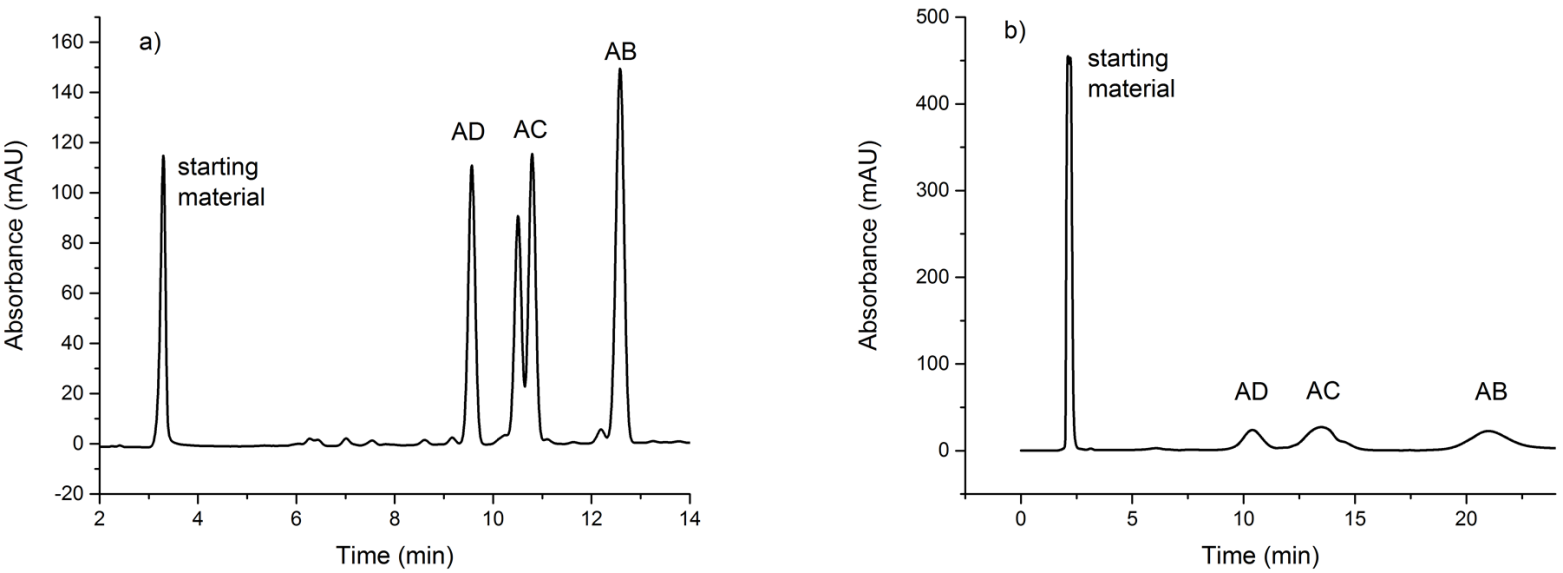
HPLC chromatograms of 6^A^-azido-6^X^-mesitylenesulfonyl-α-CD **8** (reaction 6): a) analytical and b) preparative scale at 25 °C.

Once, the regioisomer pattern has been identified (Figure 3), we developed separation methods for the 6^A^-azido-6^X^-mesitylenesulfonyl-α-CD regioisomers **8b**, **8c**, and **8d**. First, the analytical method was developed (Figure 4a) using UV–vis detection with an ACN/water gradient elution and the regioisomeric ratio of AB/AC/AD = 32:44:24. The analytical-scale method also confirmed that the mesitylenesulfonyl moiety was not hydrolyzed during the separation and that this functional group can be used to separate heterodisubstituted regioisomers in gram-scale quantities by reversed-phase column chromatography (Figure 4b). The starting material 6^A^-azido-α-CD **7**, each 6^A^-azido-6^X^-mesitylenesulfonyl-α-CD regioisomers **8b**, **8c**, **8d** and the oversubstituted 6^A^-azido-6^X^,6^Y^-dimesitylenesulfonyl-α-CD products were separated in a single, quick and reproducible separation step. Figures 4a,b also shows that the conversion to the corresponding AB, AC, AD heterodisubstituted products is incomplete due to the formation of additional substituted derivatives (6^A^-azido-6^X^,6^Y^-dimesitylenesulfonyl-α-CD) that could not be avoided. Consequently, the starting material 6^A^-azido-α-CD remains the main component of the reaction mixture and thus can be recovered. To our surprise, we observed a peak splitting (Figure 4a) for the AC regioisomer which could be caused by the presence of pseudoenantiomers (AC/CA) as will be discussed further in the section on pseudoenantiomers identification.

Pure AB, AC and AD regioisomers were isolated by reversed-phase column chromatography eluting with an ACN/H_2_O gradient. The AB **8b** and AC **8c** regioisomers were separated and obtained in the same yield 4% and 4%, whereas the AD regioisomer **8d** was obtained in 3% yield (calculated from the starting 6^A^-azido-α-CD) thus corresponding with the HPLC regioisomeric ratios. Subsequently, each regioisomer was characterized by NMR, HRMS, IR and optical rotation measurements.

### NMR characterization of heterobifunctionalized-α-CD derivatives

**Figure 5:**
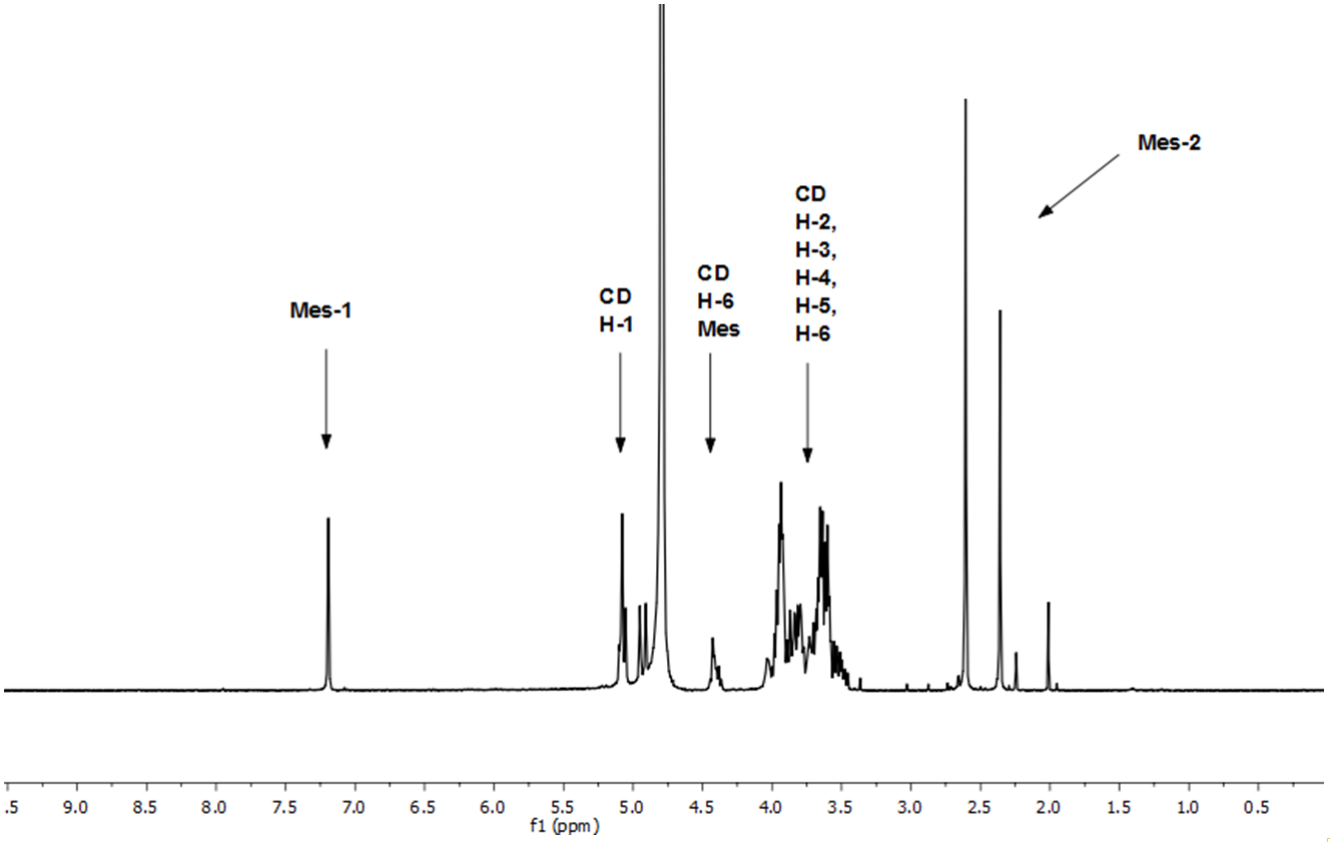
^1^H NMR spectrum of the AC regioisomer **8c** as a mixture of pseudoenantiomers prepared through reaction 6 (D_2_O, 600 MHz).

Each regioisomer separated after reaction 6 was dissolved in deuterated water (D_2_O) to simplify NMR peak assignment (assignment in DMSO-*d*_6_ included OH groups). Overall, the ^1^H NMR spectra of all three regioisomers match the disubstituted α-CD derivatives pattern. The separated AC regioisomer **8c** was chosen as a representative example (measurements were done with the mixture of pseudoenantiomers). Specifically, three basic sets of signals were observed (Figure 5). The first (Mes-1) corresponded to the mesitylene aromatic peak (7.15 ppm), the second to the α-CD region (5.24–3.41 ppm) and the third (Mes-2) to methyl groups on the mesitylene skeleton (2.60–2.30 ppm). In the CD region, three areas are well distinguished – peaks about 5.07 ppm (CD H-1) belong to H-1 protons of four unsubstituted glucose units, whereas H-1 protons of glucoses with the azido moiety and the mesitylenesulfonyl group are shifted upfield (4.98–4.95 ppm). The separated signal of the CD region refers to H-6 protons (CD H-6 Mes) attached to the mesitylenesulfonyl group (≈ 4.40 ppm).

**Figure 6:**
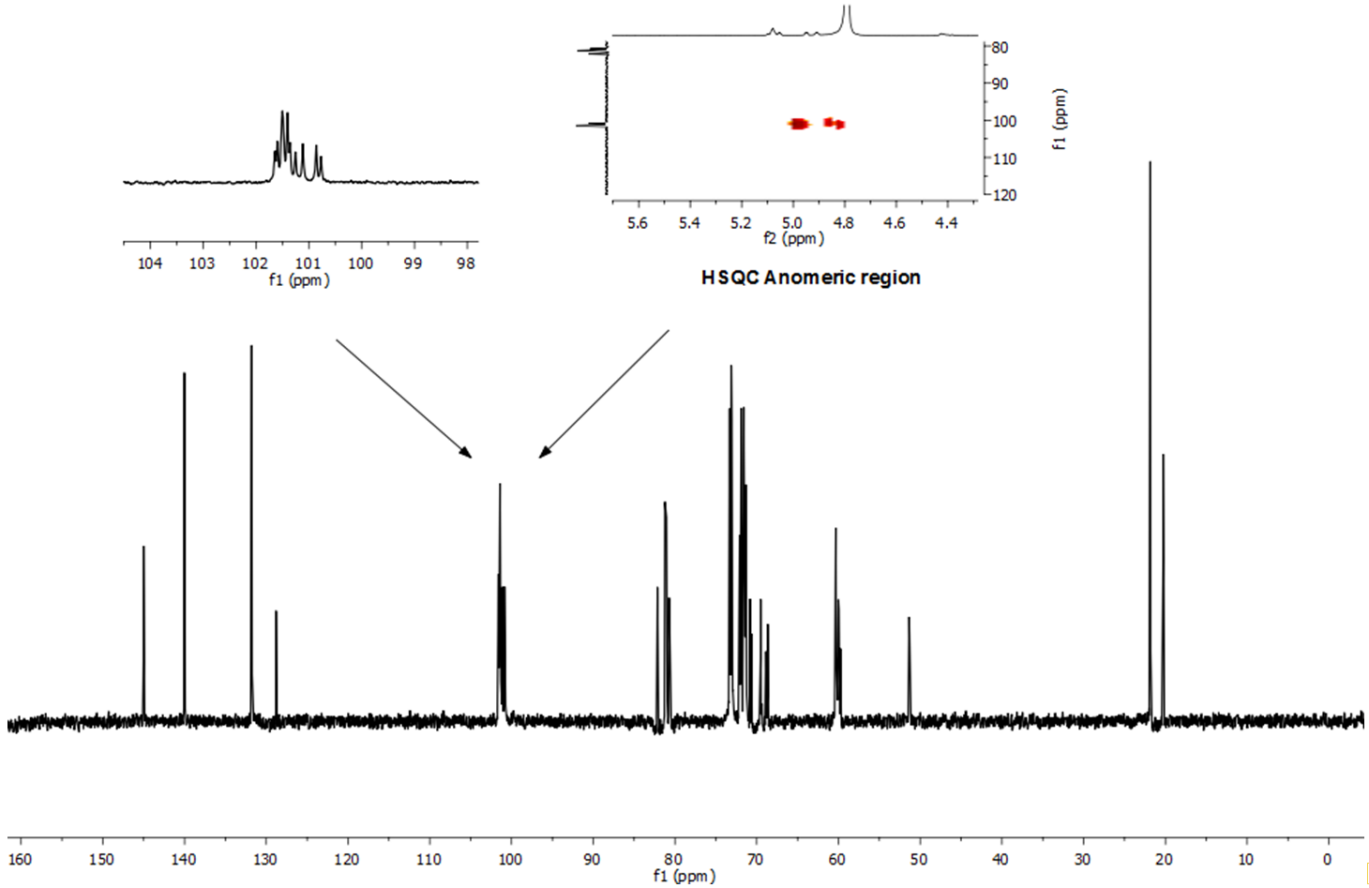
^13^C NMR spectrum of the AC regioisomer **8c** as a mixture of pseudoenantiomers prepared through reaction 6 (D_2_O, 125 MHz).

In Figure 6, the ^13^C NMR spectrum also shows the substituted C-6 carbons at 51 ppm for the azido and 68 ppm for the mesitylenesulfonyl moieties. Both substituents on the α-CD primary rim were also confirmed by HSQC in the anomeric region distinguished in three different parts. However, the crucial splitting of six C-1 anomeric doublets for the characterization of each glucose unit was not achieved.

Overall, the two different substituents (mesitylenesulfonyl and azido) are attached to the primary rim of α-CD. The incomplete anomeric doublets splitting prevented us from identifying the different regioisomeric peaks fully by NMR measurements.

### Pseudoenantiomers identification of heterodisubstituted α-CDs by HPLC–MS

**Figure 7:**
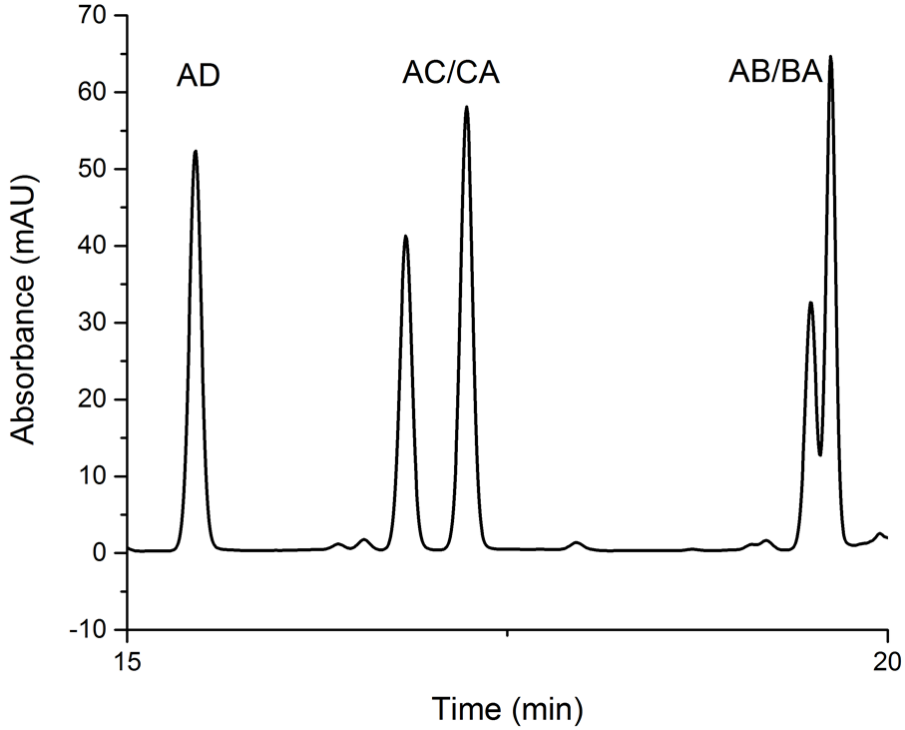
HPLC–MS chromatogram with the separated pseudoenantiomers of 6^A^-azido-6^B^-mesitylenesulfonyl-α-CD **8b** and 6^A^-azido-6^C^-mesitylenesulfonyl-α-CD **8c** at 10 °C.

Furthermore, we observed (Figure 4a) a peak splitting in the case of the AC heterodisubstituted CD regioisomer **8c** at room temperature (25 °C) during HPLC which was explained by the formation of possible pseudoenantiomers AC/CA (or AC/AE). A further HPLC–MS analysis unambiguously confirmed our hypothesis because the splitting was also observed in the case of AB regioisomer **8b** at 10 °C in the HPLC trace (Figure 7). Conversely, no peak splitting was observed in the case of the AD regioisomer **8d** as this symmetrically heterodisubstituted regioisomer could not contain a pair of pseudoenantiomers (AD/DA). The separation of the pseudoenantiomers was done on an analytical scale only. The regioisomers from preparative column chromatography were isolated as mixtures of pseudoenantiomers (in cases AB and AC). More details about the analytical-scale pseudoenantiomer separations are given in Supporting Information File 1. Our results were also compared with the studies by Sollogoub et al. [27] who did not observe pseudoenantiomers for AD heterodisubstituted α-CD derivatives. Nevertheless, our findings open a way to study pseudoenantiomers on the primary rim of heterobifunctionalized α-CD skeletons, which have remained mostly overlooked thus far.

## Conclusion

Direct, short and reproducible methods for the preparation of α-CDs derivatives homobifunctionalized on the primary rim were developed. These synthetic approaches used 6^A^,6^X^-di(aryl)sulfonyl-α-CDs, 6^A^,6^X^-dibromo-α-CDs and 6^A^,6^X^-ditosyl-α-CDs as intermediates which were transformed to the corresponding 6^A^,6^X^-diazido-α-CDs derivatives. After the preparation, homobifunctionalized regioisomers of 6^A^,6^X^-diazido-α-CDs were separated via ad hoc-developed HPLC analytical methods, and the regioisomeric patterns of these disubstituted α-CD derivatives was unambiguously identified. Furthermore, this study clarified the regioisomeric pattern of the most commonly used and key intermediates of α-CD primary rim homodisubstitution. The regioisomeric pattern was applied to the effective, large-scale separation of heterobifunctionalized 6^A^-azido-6^X^-mesitylenesulfonyl-α-CD regioisomers (as mixtures of pseudoenantiomers). These new heterobifunctionalized AB, AC, AD regioisomers of the α-CD decorated with azido and mesitylenesulfonyl functional groups can be used as key intermediates for a wide range of new α-CD derivatives. Finally, their pseudoenantiomers (AB/BA and AC/CA) were confirmed and resolved for the first time. Ultimately, these results enable the preparation of new types of pure, single isomer, pseudoenantiomer-resolved disubstituted α-CD derivatives, which may be used in a wide range of applications, particularly in organocatalysis.

## Supporting Information

File 1 Instruments, materials, detailed experimental procedures, data for each regiochemical analysis, pseudoenantiomer resolution. NMR and HRMS data of prepared compounds.
