# Supplementary material for: Enhanced single-isomer separation and pseudoenantiomer resolution of new primary rim heterobifunctionalized α-cyclodextrin derivatives
Source: Beilstein J Org Chem. 2018 Nov 13;14:2829–37. doi: 10.3762/bjoc.14.261 (PMC6244315; doi:10.3762/bjoc.14.261)
Supplement: File 1 — Instruments, materials, detailed experimental procedures, data for each regiochemical analysis, pseudoenantiomer resolution. NMR and HRMS data of prepared compounds. [file Beilstein_J_Org_Chem-14-2829-s001.pdf]

BEILSTEIN JOURNAL OF ORGANIC CHEMISTRY

Supporting Information

for

Enhanced single-isomer separation and pseudoenantiomer resolution of new primary rim heterobifunctionalized α-cyclodextrin derivatives

Iveta Tichá, Gábor Benkovics, Milo Malanga and Jindřich Jindřich

Beilstein J. Org. Chem. **2018,** *14,* 2829–2837. doi:10.3762/bjoc.14.261

Instruments, materials, detailed experimental procedures, data for each regiochemical analysis, pseudoenantiomer resolution. NMR and HRMS data of prepared compounds

License and Terms: This is a supporting information file under the terms of the Creative Commons Attribution License (http://creativecommons.org/licenses/by/4.0). Please note that the reuse, redistribution and reproduction in particular requires that the authors and source are credited.
The license is subject to the *Beilstein Journal of Organic Chemistry* terms and conditions: (https://www.beilstein-journals.org/bjoc)

Table of contents

Instruments and materials S3
Synthesis of homobifunctionalized α-CD derivatives S6
Synthesis of heterobifunctionalized α-cyclodextrins S13
References S15
^{1}H NMR and ^{13}C NMR of homo- and heterobifunctionalized α-CDs S16
2D NMR spectra of heterobifunctionalized α-CDs S23
HRMS spectra of prepared compounds.. S29

Instruments and materials

α-Cyclodextrin was the product of Wacker Chemie AG (Germany). Reagents and solvents such as *N*,*N*-dimethylformamide (DMF), pyridine, acetonitrile (ACN), methanol (MeOH), and acetone were of reagent grade and sourced from Molar Chemicals Kft (Hungary), *p*-toluenesulfonyl chloride (98%), benzene-1,3-disulfonyl chloride (97%), biphenyl-4,4'-disulfonyl chloride (97%), triphenylphosphine (99%), sodium methoxide solution in methanol (25% w/w), *N*-bromosuccinimide (NBS 99%), and 2-mesitylenesulfonyl chloride (99%) were obtained from Sigma-Aldrich (USA), and sodium azide (99%) was sourced from Merck (Germany).

Thin layer chromatography (TLC) was performed on silica gel-coated aluminum sheets DC-Alufolien Kieselgel 60 F265 (Merck, Germany). Plates were developed in a saturated chamber in eluent A: 1,4-dioxane/conc. aq. ammonia (NH_3, 25%) 10:7 (v/v) or in eluent B: 1,4-dioxane/conc. aq. ammonia (NH_3, 25%)/1-propanol 10:7:3 (v/v/v). Visualization of the CD derivatives was achieved under UV light at 254 nm and by dipping the TLC plates in 50% H_2SO_4/ethanol solution and subsequent carbonization using a heat gun. Quantitative analysis of TLC plates was performed with the software JustQuantify Free.

Preparative chromatographic separations were performed on a Büchi preparative chromatography system using SiliCycle SiliaCartridger – 40 mm Cartridge packed with Lichroprep RP-18 Phase (40–63 µm) reversed-phase silica gel as a stationary phase, ACN/water gradient elution and a Büchi UV Photometer C-635 as a detector (detection wavelengths: 280 nm for mesitylene derivatives and 214 nm for azido derivatives).

HPLC regiochemical measurements were carried out on an Agilent 1100 HPLC system equipped with a UV–vis and evaporative light scattering (ELS) detector. Reversed-phase separations were carried out on an Inertsil ODS-3 (4.6 × 150 mm, particle size 5 μm) analytical column using ACN/water as the mobile phase with gradient elution at a flow rate of 1.0 mL/min with UV monitoring (280 nm for mesitylene derivatives and 214 nm for azido derivatives). Inclusion-assisted HPLC separations were obtained on a CD-Screen stationary phase (Bio-Sol-Dex Ltd, Hungary, 4.6 × 250 mm, particle size 5 μm) with the mobile phase of ACN/water and gradient elution at a flow rate of 0.5 mL/min with UV monitoring (280 nm for mesitylene and 214 nm for azido derivatives) detection.

The pseudoenantiomers HPLC–MS measurements were carried out on a Poroshell C-18 (4.6 × 150 mm, particle size 2.7 μm) analytical column using acetonitrile (+ 0.05% formic acid)/water (+ 0.05 % formic acid) as the mobile phase. Linear gradient elution was used: 20% acetonitrile in 0 min, 30% acetonitrile at 15 min, 70% acetonitrile at 17.5 min and stop time was 25 min. Flow rate was 0.7 mL/min and the temperature was 10 °C. UV detection was done at 280nm.

Accurate mass measurements (HRMS) were obtained by ESI on an Agilent 6530Q-TOF MS spectrometer using Agilent Mass Hunter Qualitative Analysis Software B.07.00, 2014. Samples were dissolved in ACN/water.

Infrared spectroscopy was measured on Thermo Nicolet AVATAR 370 FT-IR instrument. All samples were suspended with KBr and measured using DRIFT method. Specific optical rotation was done on Rudolph Research AUTOPOL III polarimeter at 589 nm (sodium D line) and values of $[\alpha]^{25}_{D}$ are reported together with used concentration (*c*, mg/100 mL) and solvent.

NMR measurements were recorded on a Bruker AVANCE III at 600 MHz (^{1}H) and 150 MHz (^{13}C) including DEPT and 2D (H,H-COSY, HSQC and HMBC) at 298 K. All chemical shift values (δ) are reported in ppm. Samples were dissolved in D_2O and *tert*-butanol was used as internal references. Numbering of atoms for NMR spectra transcription was done according to the structures in Figure S1.

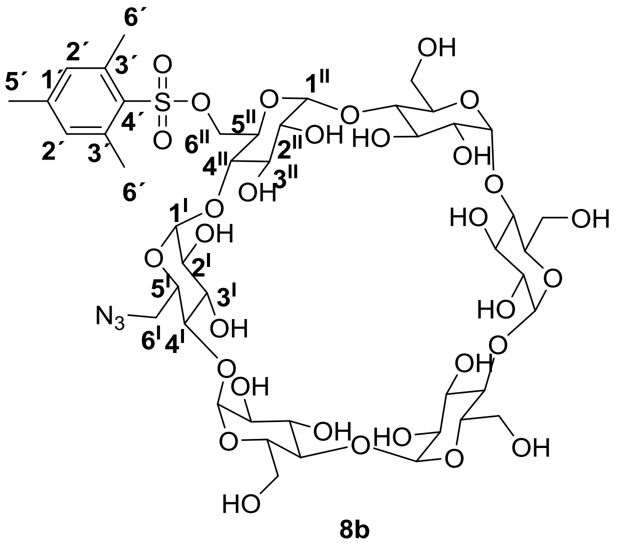

8b

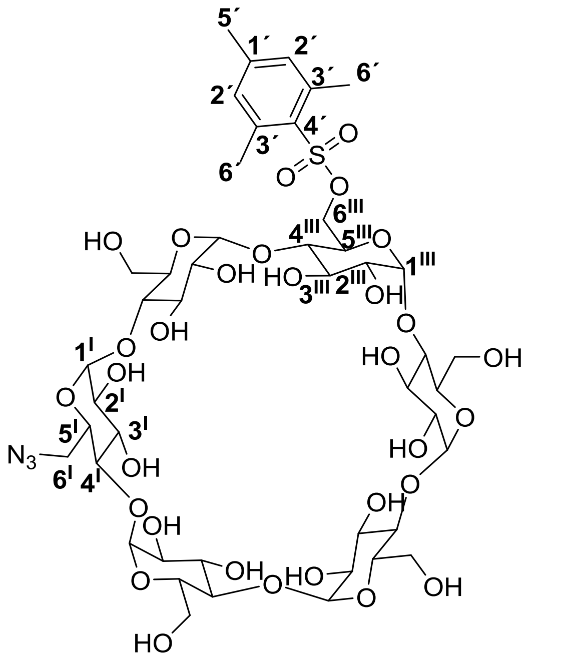

8c

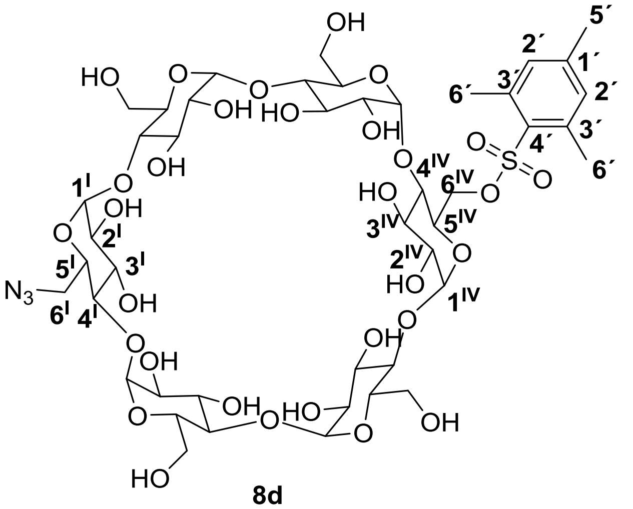

8d

Figure S1: Numbering of atoms in cyclodextrin derivative structures used for NMR.

Synthesis of homobifunctionalized α-CD derivatives

Synthesis of $6^A,6^X$-diazido-α-CDs using the “capping” methods

$6^A,6^B$-Diazido-$6^A,6^B$-dideoxy-α-cyclodextrin (4, reaction 1):

$6^A,6^B$-Capped α-CD **2** was prepared according to the modified procedure described for β-CD by Tabushi et al.[1]. Dried α-CD (**1**, 5 g, 5.1 mmol) was dissolved in freshly distilled pyridine (115 mL). *m*-Benzene-1,3-disulfonyl chloride (1.41 g, 5.1 mmol) was dissolved in freshly distilled pyridine (40 mL), and the resulting pale yellow solution was added dropwise to the α-CD solution under vigorous stirring at 8 °C within 1 hour. The mixture was stirred at 25 °C for additional 4 hours. It was observed white precipitation after finishing of the stirring. The white precipitate (part of unreacted α-CD) was filtered off, and the mother liquid was evaporated under reduced pressure at 30 °C. The remaining gel-like, pale yellow residue was subsequently poured into 200 mL of MeOH resulting in the formation of a white precipitate. The solid was recovered by filtration, washed with MeOH (3 × 50 mL) and dried to constant weight in a vacuum drying box in the presence of P_2O_5 and KOH.

The solid (0.87 g), containing unreacted α-CD (≈ 90% based on TLC, R_f = 0.35, eluent A) and $6^A,6^B$-capped α-CD (**2**, ≈10% based on TLC, R_f = 0.5, eluent A), was dissolved in DMF (20 mL), sodium azide (0.14 g, 2.2 mmol) was added and the mixture was heated to 80 °C for 5 hours. DMF was removed under reduced pressure at 60 °C and the yellowish residue was poured into acetone (200 mL) under vigorous stirring. The formed white precipitate was recovered by filtration, washed with acetone (3 × 50 mL) and dried to constant weight (0.84 g) in a vacuum drying box in the presence of P_2O_5 and KOH. Direct-phase TLC (eluent A) and reversed-phase

HPLC (ACN/H_2O gradient elution) analysis revealed that the precipitate contained unreacted α-CD (**1**, R_f = 0.35), monoazido-α-CD (**7**, R_f = 0.5) and diazido-α-CD (**4**, R_f = 0.65). The diazido-α-CD fraction was isolated by direct column chromatography. The precipitate was dissolved in ACN/water/NH_3 (25%) 10:7:1 (8.7 mL) and injected into the chromatographic column (44 g). The mobile phase was ACN/water/NH_3 (25%) 10:5:1. After chromatographic separation, the 6^A,6^B-diazido-α-CD (**4**, 0.09 g, 2% yield) was obtained as a white solid. 6^A-Azido-α-CD (**7**) was also obtained as a white solid (0.19 g, 4% yield) as the second eluted compound.

6^A,6^X-Diazido-6^A,6^X-dideoxy-α-cyclodextrin (**4**, reaction 2):

6^A,6^X-Capped α-CD **3** was prepared according to the modified procedure described for β-CD by Tabushi et al.[2]. Dried α-CD (**1**, 5 g, 5.1 mmol) was dissolved in freshly distilled pyridine (77 mL). The mixture was heated to 50 °C and biphenyl-4,4´-disulfonyl chloride (1.4 g, 3.8 mmol) was added during one hour in four portions in 15 minutes interval. The resulting yellow solution was stirred at 50 °C for additional 1.5 h and then evaporated under reduced pressure at 25 °C. The gel-like, pale yellow residue was subsequently poured into MeOH (200 mL) resulting in the formation of a white precipitate. The solid was recovered by filtration, washed with methanol (3 × 50 mL) and dried to constant weight in a vacuum drying box in the presence of P_2O_5 and KOH.

This white solid (3.27 g), containing unreacted α-CD (**1**, ≈ 85% based on TLC, R_f = 0.35, eluent A) and 6^A,6^X-capped α-CD (**3**, ≈ 15% based on TLC, R_f = 0.55, eluent A), was dissolved in DMF (30 mL). Sodium azide (0.34 g, 5.2 mmol) was added, and the reaction mixture was heated to 80 °C for 5 h. Unreacted NaN_3 was filtered off, the reaction mixture was concentrated under reduced pressure at 50 °C and the yellowish residue was poured into acetone (220 mL) under vigorous stirring. The

white precipitate was recovered by filtration, washed with acetone (3 × 50 mL) and dried to constant weight (2.45 g) in a vacuum drying box in the presence of P_2O_5 and KOH. Direct-phase TLC (eluent A) and reversed-phase HPLC (ACN/H_2O gradient elution) analysis revealed that the precipitate contained unreacted α-CD (**1**, R_f = 0.35), monoazido-α-CD (**7**, R_f = 0.5) and diazido-α-CD (**4**, R_f = 0.65). The diazido-α-CD fraction was isolated by direct column chromatography. The precipitate was dissolved in ACN/water/NH_3 (25%) 10:7:1 (24 mL) and injected into the chromatographic column (134 g). The mobile phase was ACN/water/NH_3 (25%) 10:5:1. After the chromatographic separation, the $6^A,6^X$-diazido-α-CD (**4**, 0.25 g, 5% yield) was obtained as a white solid material. 6^A-Azido-α-CD (**7**) was also obtained as a white solid (0.34 g, 7% yield) as the second eluted compound.

Synthesis of $6^A,6^X$-diazido-α-CDs *via* dibromo-α-CD intermediates

$6^A,6^X$-Diazido-$6^A,6^X$-dideoxy-α-cyclodextrin (4, reaction 3):

$6^A,6^X$-Brominated α-CD **5** was prepared according to the modified procedure [3]. Triphenylphosphine (36.4 g, 138 mmol) was dissolved in 125 mL of freshly distilled DMF under Ar atmosphere. The solution was cooled down to 8 °C, and NBS was slowly added to the solution which became dark violet. Dried α-CD (**1**, 25 g, 25.7 mmol) was dissolved in 100 mL of DMF and added in one portion during 20 minutes to the dark violet reaction mixture. The mixture was heated up to 50 °C, and the conversion to dibromo-α-CD was monitored by direct phase TLC (eluent B). After 7.5 h the reaction was stopped by addition of 100 mL of MeOH. After 10 minutes of vigorous stirring, the red-violet reaction mixture was cooled and poured to 1.3 mL of MeOH (pH 3). Addition of sodium methoxide solution (100 mL) resulted in slow precipitation (pH 12). The yellowish precipitate was recovered by filtration, washed

with MeOH (3 × 300 mL) and dried to constant weight in a vacuum drying box in the presence of P_2O_5 and KOH.

The white solid (15.27 g), containing unreacted α-CD (**1**, ≈ 35% based on TLC, R_f = 0.15, eluent B), monobromo-α-CD (≈ 35% based on TLC, R_f = 0.25, eluent B), dibromo-α-CD (**5**, ≈ 20% based on TLC, R_f = 0.35, eluent B) and oversubstituted products (less than 10% based on TLC, R_f = 0.5, eluent B) was dissolved in DMF (300 mL), sodium azide (2.72 g, 42.0 mmol) was added, and the mixture was heated to 80 °C for 5 h. DMF was removed under reduced pressure at 60 °C and the yellowish residue was poured into acetone (1.3 L) under vigorous stirring. The white precipitate was recovered by filtration, washed with acetone (3 × 100 mL) and dried to constant weight (17.9 g) in a vacuum drying box in the presence of P_2O_5 and KOH. Direct-phase TLC (eluent B) and reversed-phase HPLC (ACN/H_2O gradient elution) analysis revealed that the precipitate contained unreacted α-CD (**1**, R_f = 0.15), monoazido-α-CD (**7**, R_f = 0.35) and diazido-α-CD (**4**, R_f = 0.45). The diazido-α-CD fraction was isolated by direct column chromatography. One-third of the precipitate was dissolved in ACN/water/NH_3 (25%) 10:7:1 (40 mL) and injected to the chromatographic column (310 g). After chromatographic separation of all three portions of the crude mixture using mobile phase ACN/water/NH_3 (25%) 10:5:1, the $6^A,6^X$-diazido-α-CD **4** (1.26 g, 5% yield) was obtained as a white solid material. 6^A-Azido-α-CD **7** was also obtained as a white solid (3.69 g, 15% yield) as the second eluted compound.

$6^A,6^D$-Diazido-$6^A,6^D$-dideoxy-α-cyclodextrin (**4d**, reaction 4):

$6^A,6^D$-Dibromo-α-CD **5d** was prepared according to the procedure by Sinaÿ et al. [4], Kraus et al. [5] and Sollogoub et al. [6]. The white solid $6^A,6^D$-dibromo-α-CD **5d** (0.05

g, 0.4 mmol) was dissolved in freshly distilled DMF (1 mL), sodium azide (0.02 g, 0.2 mmol) was added, and the reaction mixture was heated to 80 °C for 5 h. DMF was removed under reduced pressure at 60 °C and the yellowish residue was poured into acetone (25 mL) under vigorous stirring. The white precipitate was recovered by filtration, washed with acetone (3 × 50 mL) and dried to constant weight in a vacuum drying box in the presence of P_2O_5 and KOH. The 6^A,6^D-diazido-α-CD was obtained as a white solid material (**4**, 0.04 g, 95% yield, R_f = 0.65, eluent A) without a need to purify on column chromatography.

Synthesis of 6^A,6^X-diazido-α-CDs using tosylation

6^A,6^X-Diazido-6^A,6^X-dideoxy-α-cyclodextrin (4, reaction 5):

6^A,6^X-Ditosyl-α-CD **6** was prepared according to the modified procedure for monotosyl-α-CD [7]. Dried α-CD (**1**, 5 g, 5 mmol) was dissolved in freshly distilled pyridine (150 mL), cooled to 0 °C and *p*-toluenesulfonyl chloride (3.49 g, 18 mmol) was added gradually spoon by spoon. After addition of *p*-toluenesulfonyl chloride, the reaction mixture was stirred at room temperature for 6 h, and then pyridine was evaporated under reduced pressure at 30 °C. The gel-like, light yellow residue obtained was then dissolved in MeOH (30 mL) and subsequently poured into acetone (350 mL), resulting in the immediate formation of a white precipitate. The solid was recovered by filtration, washed with acetone (3 × 50 mL) and dried to constant weight in a vacuum drying box in the presence of P_2O_5 and KOH.

The white material (6.3 g), containing unreacted α-CD (**1**, ≈ 35% based on TLC, R_f = 0.15, eluent B), 6^A-tosyl-α-CD (≈ 30% based on TLC, R_f = 0.3, eluent B), 6^A,6^X-ditosyl-α-CD (**6**, ≈ 30% based on TLC, R_f = 0.45, eluent B) and the overtosylated

$6^A,6^X,6^Y$-tritosyl-α-CD (≈ 20% based on TLC, R_f = 0.55, eluent B) was dissolved in DMF (120 mL), sodium azide (0.76 g, 17 mmol) was added and the reaction mixture was heated to 80 °C for 5 hours. DMF was removed under reduced pressure at 60 °C and the yellowish residue was poured into acetone (300 mL) under vigorous stirring. The white precipitate was recovered by filtration, washed with acetone (3 × 50 mL) and dried to constant weight (5.74 g) in a vacuum drying box in the presence of P_2O_5 and KOH. Direct-phase TLC (eluent B) and reversed-phase HPLC (ACN/H_2O gradient elution) analysis revealed that the precipitate contained unreacted α-CD (**1**, R_f = 0.15), monoazido-α-CD (**7**, R_f = 0.35) and diazido-α-CD (**4**, R_f = 0.45) and triazido-α-CD (R_f = 0.5). The diazido-α-CD fraction was isolated by direct column chromatography (394 g). The precipitate was dissolved in ACN/water/NH_3 (25%) 10:7:1 (50 mL) and injected to the chromatographic column. After chromatographic separation using mobile phase ACN/water/NH_3 (25%) 10:5:1, the $6^A,6^X$-diazido-α-CD (**4**, 0.69 g, 16% yield) was obtained as a white solid material. 6^A-Azido-α-CD was also obtained as a white solid (**7**, 0.46 g, 11% yield) as the second eluted compound.

Synthesis of $6^A,6^X$-diazido-α-CDs from 6^A-azido-6^X-mesitylenesulfonyl-α-CD

$6^A,6^X$-Diazido-$6^A,6^X$-dideoxy-α-cyclodextrin (4, reaction 7):

$6^A,6^X$-Diazido-α-CD was prepared from 6^A-azido-6^X-mesitylenesulfonyl-α-CD (see procedure below) following classical azidation conditions. 6^A-Azido-6^X-mesitylenesulfonyl-α-CD (**8**, 0.3 g, 0.2 mmol) was dissolved in freshly distilled DMF (5 mL), sodium azide (0.044 g, 1.9 mmol) was added, and the reaction mixture was heated to 80 °C for 5 h. DMF was removed under reduced pressure at 60 °C

and the yellowish residue was poured to acetone (300 mL) under vigorous stirring. The white precipitate was recovered by filtration, washed with acetone (3 × 50 mL) and dried to constant weight (0.2 g) in a vacuum drying box in the presence of P_2O_5 and KOH. Direct-phase TLC (eluent B) and reversed-phase HPLC (ACN/H_2O gradient elution) analysis revealed that the precipitate contained 6^A-azido-α-CD (**7**, ≈ 60% based on TLC, R_f = 0.35) and $6^A,6^X$-diazido-α-CD (**4**, ≈ 35% based on TLC, R_f = 0.45) and more substituted $6^A,6^X,6^Y$-triazido-α-CD (≈ 5% based on TLC, R_f = 0.5). The $6^A,6^X$-diazido-α-CD fraction was not isolated by direct column chromatography and used directly as a mixture of starting material, diazido-α-CD and triazido-α-CD in HPLC separations.

^{1}H NMR, ^{13}C NMR spectra for diazido-α-CDs were measured (i) as a mixture of three regioisomers (ii) as a single AD isomer (Figure S2–S4) and are in accordance with literature [6].

^{1}H NMR (600 MHz, D_2O, 298 K): δ (ppm): 5.07 (m, H-1, 6H), 4.02 –3.82 (m, 24H, H-3, H-5, H-6), 3.71 –3.57 (m, 12H, H-2, H-4). ^{13}C NMR (150 MHz, D_2O, 298 K): δ (ppm): 103.99, 103.81, 84.73, 83.90, 75.87, 75.65, 74.71, 74.51, 74.17, 74.10, 73.29, 63.00, 53.80.

HRMS data are identical for all homobifunctionalized $6^A,6^X$-diazido-α-CDs. HRMS: $[M+Na]^+$ found 1045.3094. For $C_{36}H_{58}N_6O_{28}$ $[M+Na]^+$ calculated 1045.3191.

IR (KBr) ν = 3354, 3321, 2104, 1416, 1332, 1293, 1655, 1207, 1033 cm^{-1}.

(i) $[\alpha]_D^{25}$ = + 90.3° (c = 0.30, H_2O) (ii) $[\alpha]_D^{25}$ = + 133.3° (c = 0.31, H_2O)

Synthesis of heterobifunctionalized α-cyclodextrins

6^A-Azido-6^A-deoxy-6^X-mesitylenesulfonyl-α-cyclodextrin (8, reaction 6):

6^A-Azido-α-CD **7** [6], was obtained as a byproduct in reaction 3. Dried 6^A-azido-α-CD (**7**, 1.23 g, 1.2 mmol) was dissolved in pyridine (12 mL) and cool to 0 °C. 2-Mesitylenesulfonyl chloride (0.52 g, 2.4 mmol) was added spoon by spoon. The reaction mixture was stirred at room temperature for 2 hours and the yellowish residue was poured to acetone (300 mL) under vigorous stirring. The white precipitate was recovered by filtration, washed with acetone (3 × 50 mL) and dried to constant weight (1.60 g) in a vacuum drying box in the presence of P_2O_5 and KOH. Direct-phase TLC (eluent B) and reversed-phase HPLC (H_2O/ACN gradient elution) analysis revealed that the precipitate contained unreacted 6^A-azido-α-CD (**7**, ≈ 60% based on TLC, R_f = 0.35, eluent B), 6^A-azido-6^X-mesitylenesulfonyl-α-CD (**8**, ≈ 35% based on TLC, R_f = 0.5, eluent B) and 6^A-azido-6^X,6^Y-dimesitylenesulfonyl-α-CD (≈ 5% based on TLC, R_f = 0.6, eluent B). The 6^A-azido-6^X-mesitylenesulfonyl-α-CD fraction was isolated by reversed-phased column chromatography (H_2O/ACN gradient elution). The first eluted (95:5), starting material, 6^A-azido-α-CD (520 mg) was recovered. Then, 6^A-azido-6^X-mesitylenesulfonyl-α-CD was eluted (70:30): AD regioisomer in 10.0 min, AC regioisomer in 13.5 min and AB regioisomer in 19.5 min. The isolated yield of regioisomers was 26.4 mg (3%) for AD **8d**, 37.5 mg (4%) for AC **8c** and 38.2 mg (4%) for AB **8b**, obtained from 960 mg precipitate of reaction mixture.

6^A-Azido-6^A-deoxy-6^D-*O*-mesitylenesulfonyl-α-cyclodextrin (8d)

^{1}H NMR (600 MHz, D_2O, 298 K): δ (ppm): 7.19 (2H, s, C-2´), 5.24 (1H, m, H-1-I), 5.08 (4H, m, H-1), 4.95 (1H, m, H-1-IV), 4.39 (2H, s, H-6-IV), 4.04 (1H, m, H-5-IV), 3.95-3.41 (34H, m, H-5, H-6, H-3, H-4, H-2), 2.60 (6H, s, CH_3-6´), 2.34 (3H, s, CH_3-5´). ^{13}C NMR (150 MHz, D_2O, 298 K): δ (ppm): 144.74 (C-1´), 140.07 (2 × C-3´), 131.67 (2 × C-2´), 128.73 (C-4´), 101.51 (5 × C-1), 99.36 (1× C-1-I), 81.11 (6 × C-4), 73.14 (6 × C-3), 72.10 (4 × C-5, 5 × C-2), 70.50 (1 × C-2-I), 69.97 (1 × C-5-IV), 68.82 (1 × C-6-IV), 60.18 (4 × C-6), 58.37 (1 × C-5-I), 51.36 (1 × C-6-I), 21.93 (2 × CH_3-6´), 20.32 (CH_3-5´). HRMS: $[M+Na]^+$, found: 1202.3444. For $C_{45}H_{69}N_3O_{31}S$ $[M+Na]^+$ calculated 1202.3528. IR (KBr) ν = 3294, 2929, 2101, 1461, 1347, 1287, 1156, 1018 cm^{-1}. $[\alpha]_D^{25}$ = + 124.6° (c = 0.25, DMSO).

6^A-Azido-6^A-deoxy-6^C-*O*-mesitylenesulfonyl-α-cyclodextrin (8c)

^{1}H NMR (600 MHz, D_2O, 298 K): δ (ppm): 7.19 (2H, s, C-2´), 5.08 (4H, m, H-1), 4.98 (1H, m, H-1-I), 4.95 (1H, m, H-1-III), 4.40 (2H, m, H-6-III), 4.03-3.72 (20H, m, H-2, H-5, H-6, H-3), 3.73-3.44 (14H, m, H-6-I, H-4), 2.59 (6H, s, CH_3-6´), 2.30 (3H, s, CH_3-5´). ^{13}C NMR (150 MHz, D_2O, 298 K): δ (ppm): 144.74 (C-1´), 140.07 (2 × C-3´), 131.67 (2 × C-2´), 128.73 (C-4´), 101.51 (5 × C-1), 100.08 (1 × C-1-I), 81.59 (6 × C-4), 73.34 (6 × C-3), 71.90 (4 × C-5, 5 × C-2), 70.83 (1 × C-2-I), 69.59 (1 × C-5-III), 68.60 (1 × C-6-III), 60.23 (4 × C-6), 51.33 (1 × C-6-I), 21.93 (2 × CH_3-6´), 20.32 (CH_3-5´). HRMS: $[M+Na]^+$, found: 1202.3435. For $C_{45}H_{69}N_3O_{31}S$ $[M+Na]^+$ calculated 1202.3528. IR (KBr) ν = 3294, 2929, 2101, 1721, 1335, 1237, 1153, 1078 cm^{-1}. $[\alpha]_D^{25}$ = + 107.9° (c = 0.26, DMSO).

6^A-Azido-6^A-deoxy-6^B-*O*-mesitylenesulfonyl-α-cyclodextrin (8b)

^{1}H NMR (600 MHz, D_2O, 298 K): δ (ppm): 7.19 (2H, s, H-2´), 5.07 (4H, m, H-1), 4.98 (1H, m, H-1-I), 4.95 (1H, s, H-1-II), 4.40 (2H, m, H-6-II), 4.03-3.72 (20H, m, H-2, H-5, H-6, H-3), 3.66-3.45 (14H, m, H-6-I, H-4), 2.60-2.59 (6H, s, CH_3-6´), 2.34 (3H, s, CH_3-5´). ^{13}C NMR (150 MHz, D_2O, 298 K): δ (ppm): 144.74 (C-1´), 140.07 (2 × C-3´), 131.67 (2 × C-2´), 128.73 (C-4´) 101.51 (6 × C-1), 81.05 (6 × C-4), 73.14 (6 × C-3), 72.10 (4 × C-2, 5 × C-2), 70.65 (1 × C-2-I), 68.60 (1 × C-5-II), 60.18 (4 × C-6), 51.10 (1 × C-6-I), 21.95-21.89 (2 × *C*H_3-6´), 20.32 (*C*H_3-5´). HRMS: $[M+Na]^+$, found: 1202.3432. For $C_{45}H_{69}N_3O_{31}S$ $[M+Na]^+$ calculated 1202.3528. IR (KBr) ν = 3294, 2929, 2104, 1419, 1350, 1242, 1150, 1030 cm^{-1}. $[\alpha]_D^{25}$ = + 95.0° (c = 0.25, DMSO).

References

1. Tabushi, I.; Nabeshima, T.; Fujita, K.; Matsunaga, A.; Imoto, T. *J. Org. Chem.* **1985**, *50*, 2638–2643.

2. Tabushi, I.; Yamamura, K.; Nabeshima, T. *J. Am. Chem. Soc.* **1984**, *106*, 5267–5270.

3. Heck, R.; Jicsinszky, L.; Marsura, A. *Tetrahedron Lett.* **2003**, *44*, 5411–5413.

4. Pearce, A. J.; Sinaÿ, P. *Angew. Chem.* **2000**, *39*, 3610–3612.

5. Kumprecht, L.; Buděšínský, M.; Vondrášek, J.; Vymětal, J.; Černý, J.; Císařová, I.; Brynda, J.; Herzig, V.; Koutník, P.; Závada, J.; Kraus, T. *J. Org. Chem.* **2009**, *74*, 1082–1092.

6. Fredy, J. W.; Scelle, J.; Guenet, A.; Morel, E.; Adam de Beaumais, S.; Ménand, M.; Marvaud, V.; Bonnet, C. S.; Tóth, E.; Sollogoub, M.; Viviers, G.; Hasenknopf, B. *Chem. - Eur. J.* **2014**, *20*, 10915–10920.

7. Tang, W.; Ng, S.-C. *Nat. Protoc.* **2008**, *3*, 691–697.

^{1}H NMR and ^{13}C NMR of homo- and heterobifunctionalized α-CDs

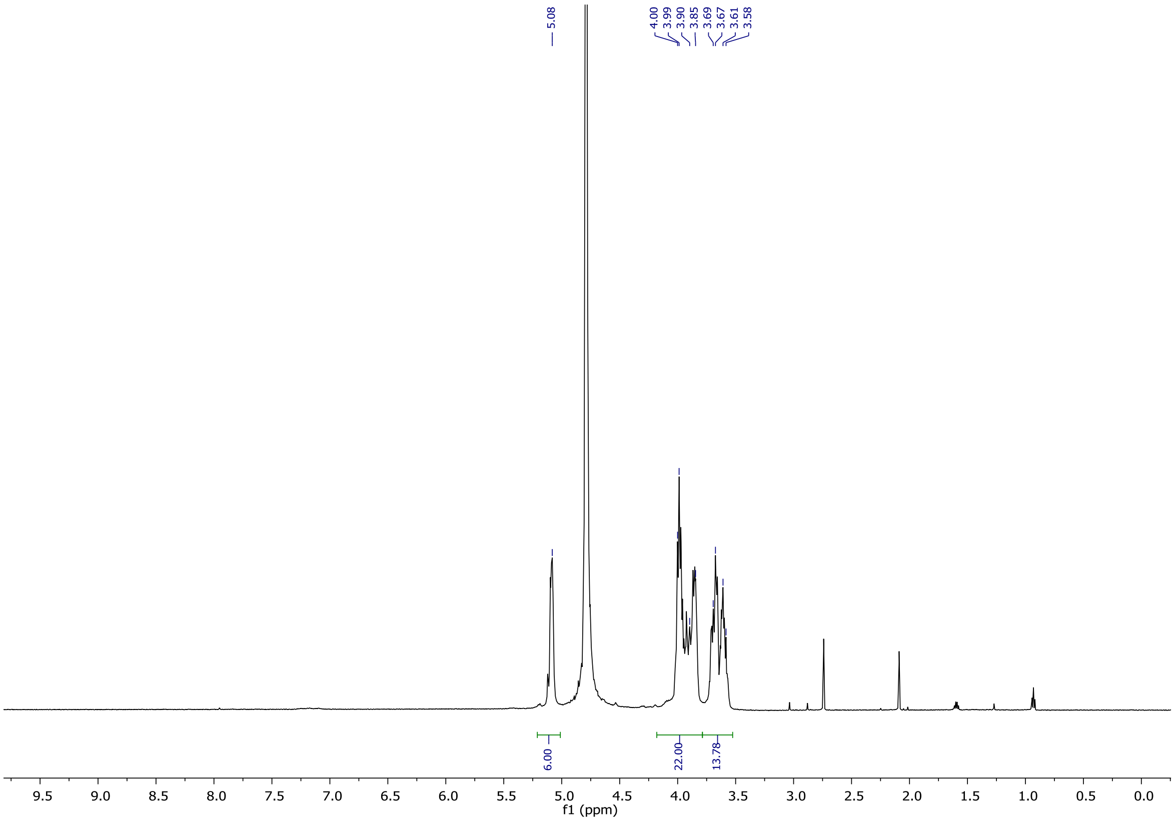

Figure S2: ^{1}H NMR spectrum of $6^{A},6^{X}$-diazido-α-CD **4** from Reaction 3 (mixture of regioisomers, D_2O, 298 K).

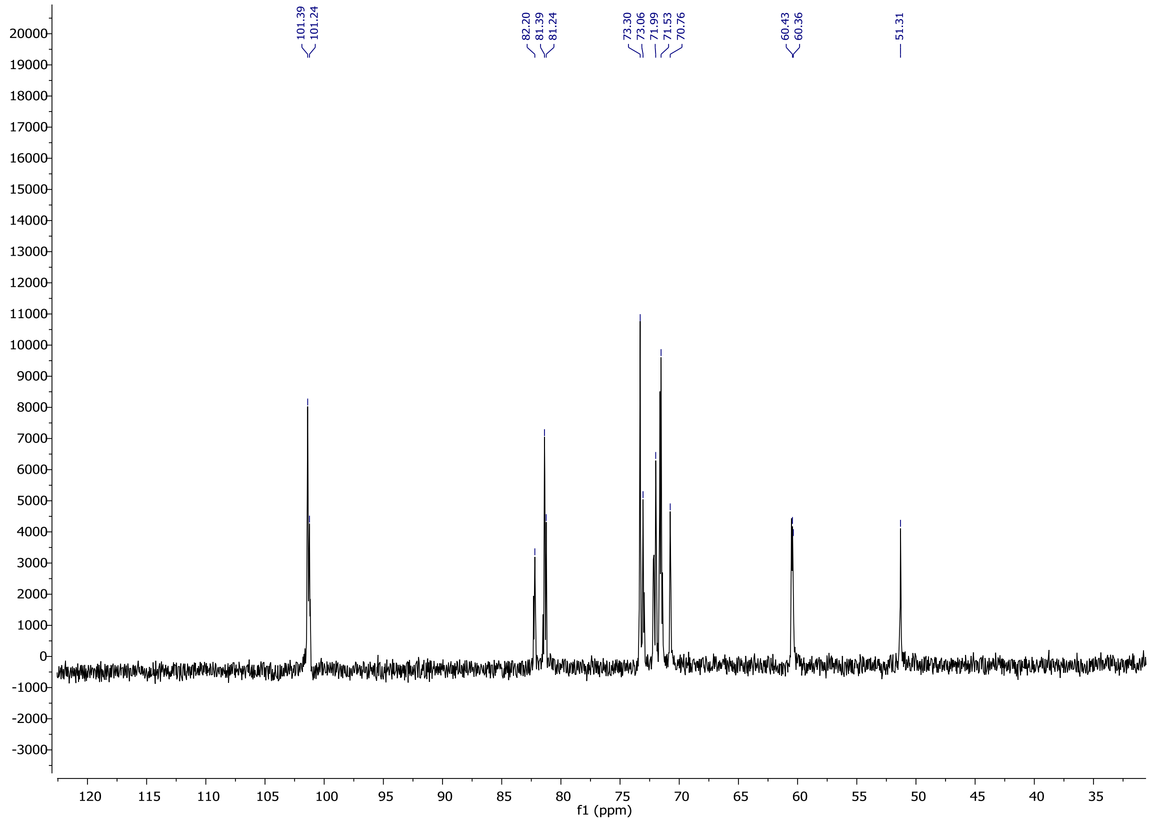

Figure S3: ^{13}C NMR spectrum of $6^{A},6^{X}$-diazido-α-CD **4** from Reaction 3 (mixture of regioisomers, D_2O, 298 K).

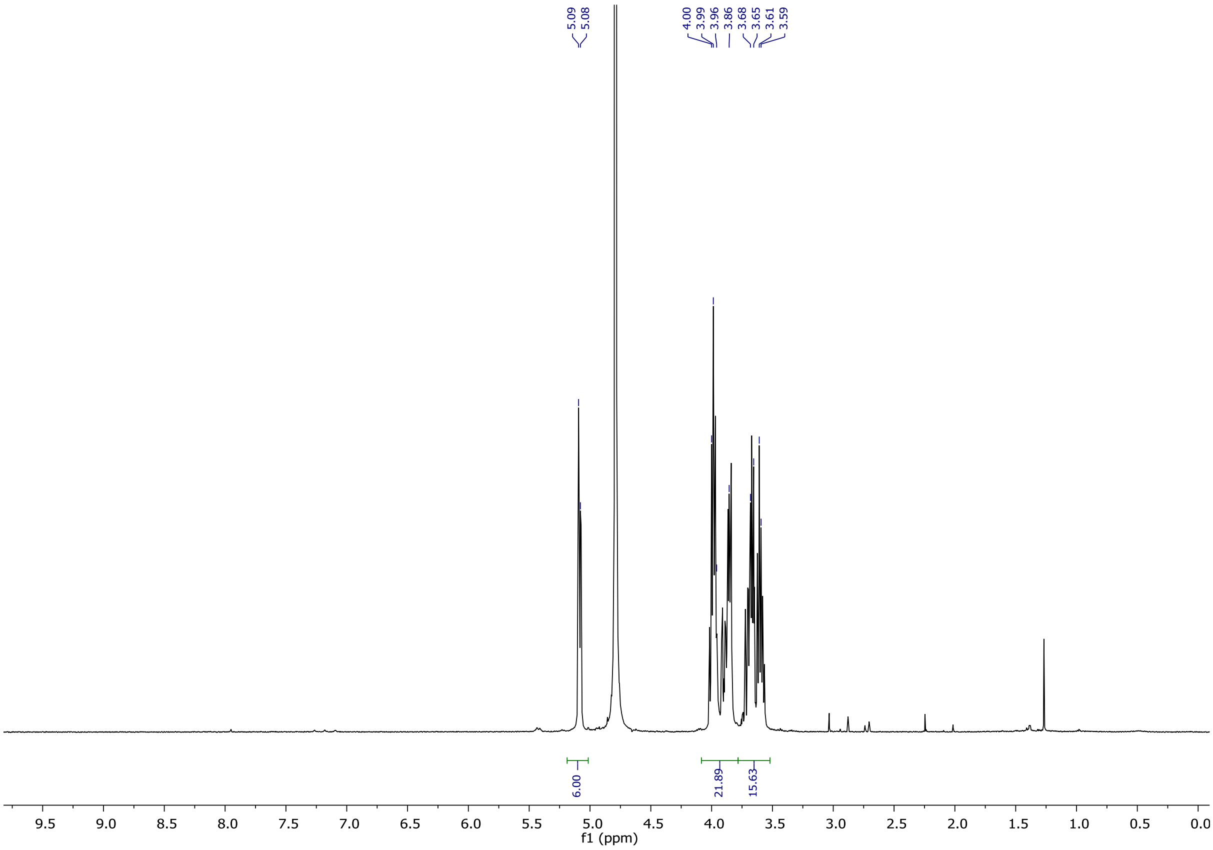

Figure S4: ^{1}H NMR spectrum of $6^{A},6^{D}$-diazido-α-CD **4d** from Reaction 4 (AD regioisomer, D_2O, 298 K).

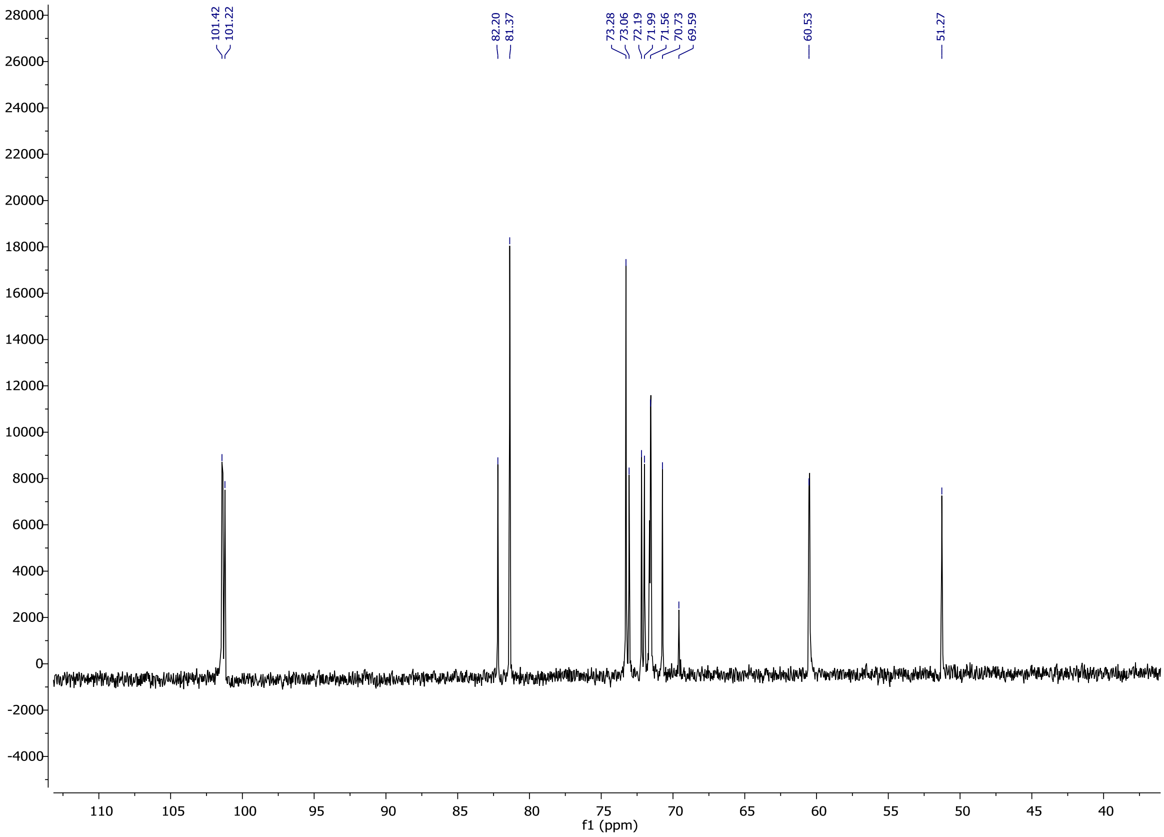

Figure S5: ^{13}C NMR spectrum of $6^{A},6^{D}$-diazido-α-CD **4d** from Reaction 4 (AD regioisomer, D_2O, 298 K).

20

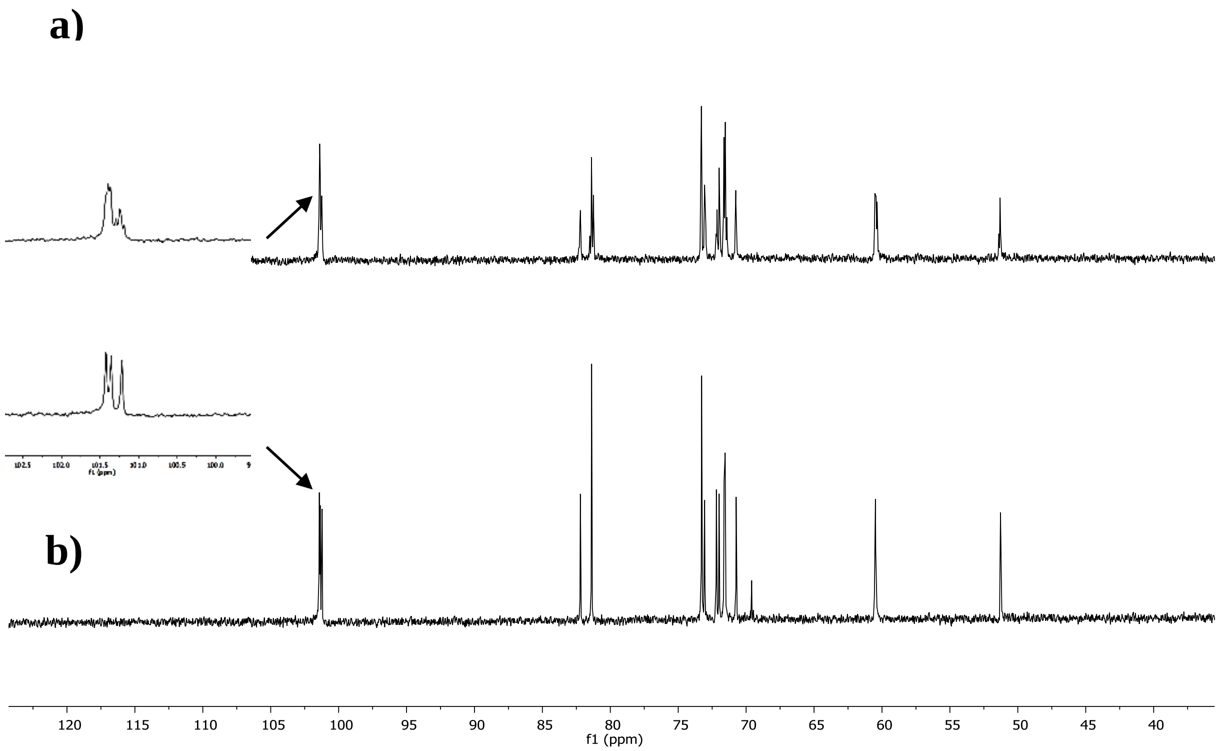

Figure S6: Comparison of ^{13}C NMR of $6^A,6^X$-diazido-α-CD **4** (mixture of regioisomers) from Reaction 3 a) (D_2O, 298 K) and $6^A,6^D$-diazido-α-CD **4d** (AD regioisomer) from Reaction 4 b) (D_2O, 298 K) and detail on C-1 carbons of glucose units.

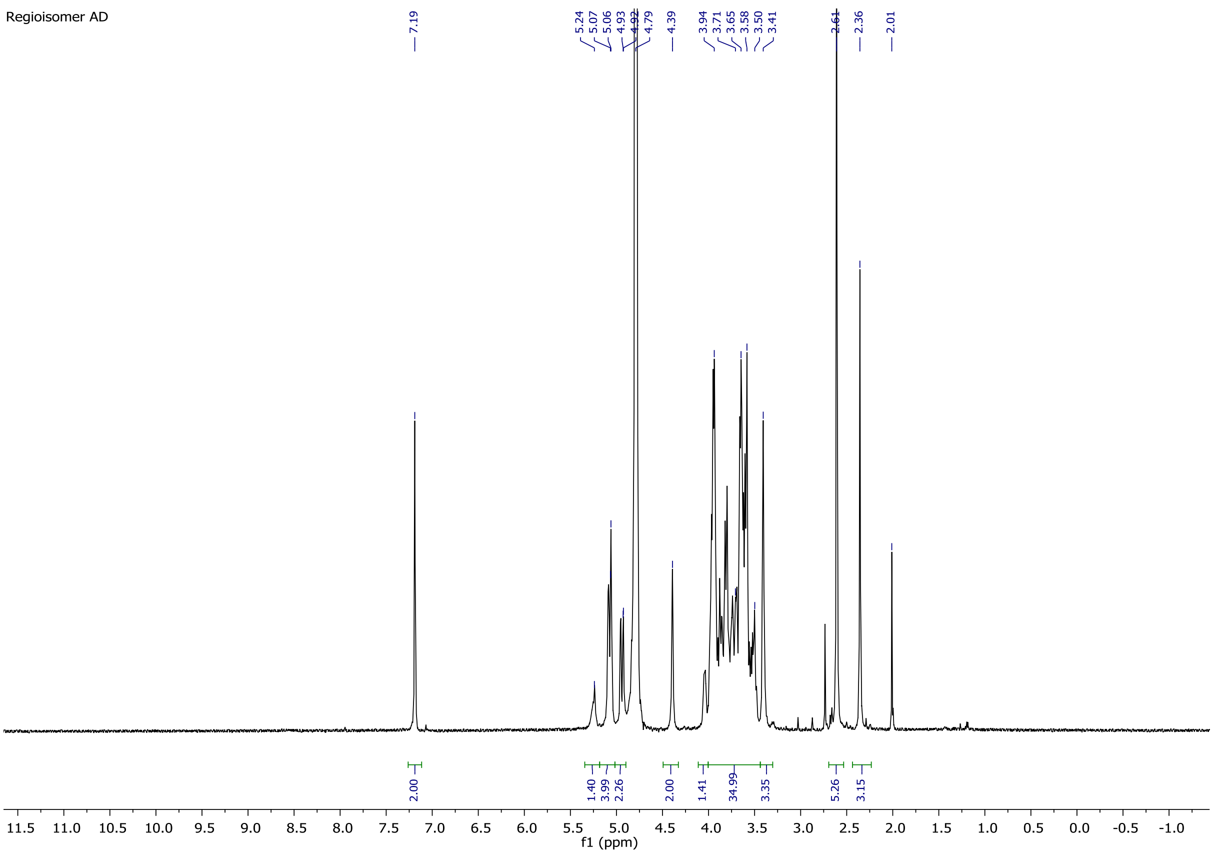

Figure S7: ^{1}H NMR spectrum of 6^{A}-azido-6^{D}-mesitylenesulfonyl-α-CD **8d** (D_2O, 298 K).

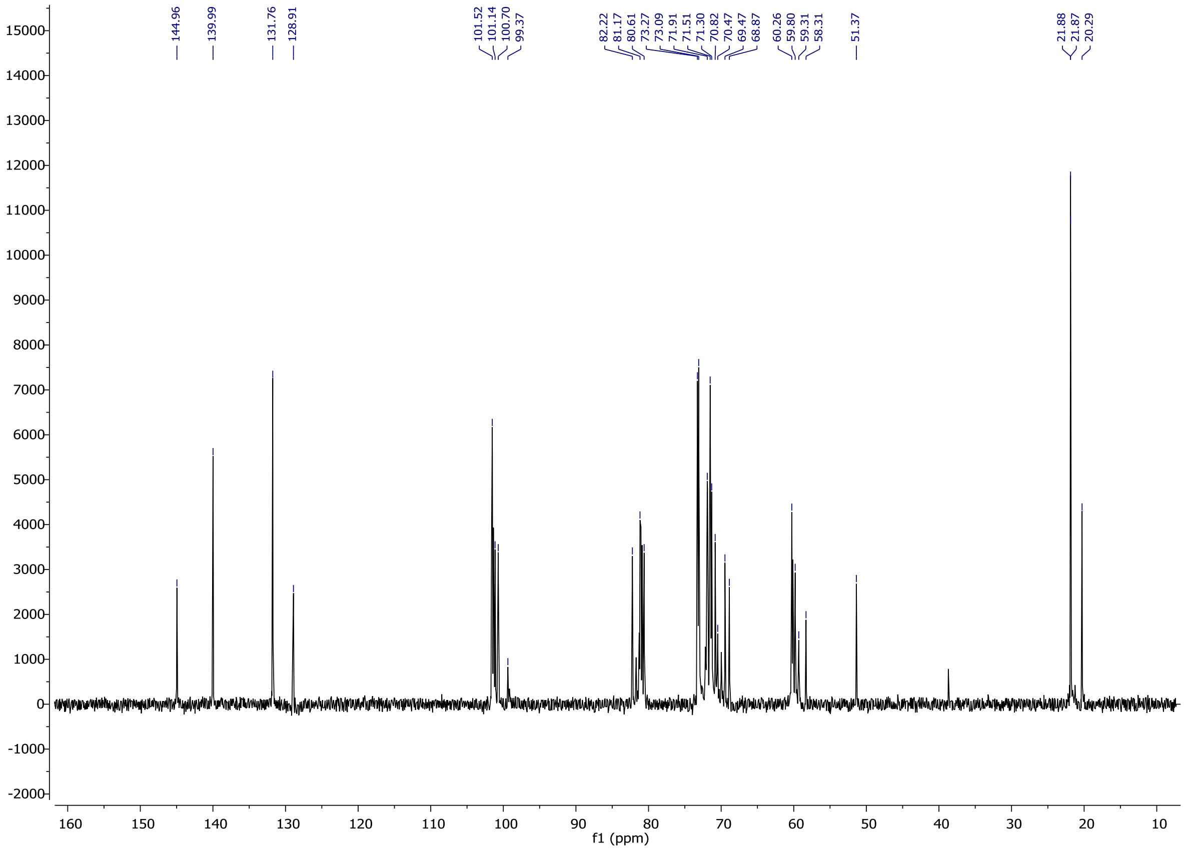

Figure S8: ^{13}C NMR spectrum of 6^{A}-azido-6^{D}-mesitylenesulfonyl-α-CD **8d** (D_2O, 298 K).

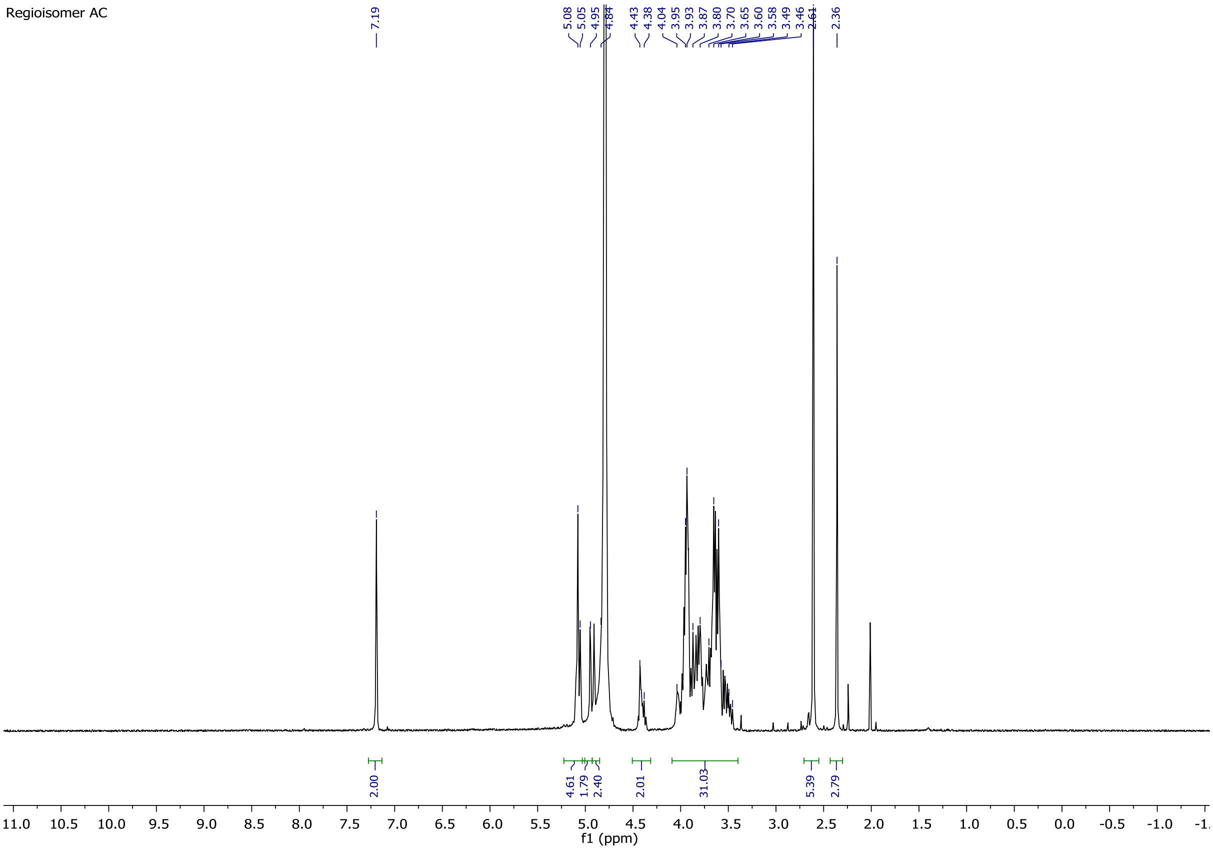

Figure S9: ^{1}H NMR spectrum of 6^{A}-azido-6^{C}-mesitylenesulfonyl-α-CD **8c** (D_2O, 298 K).

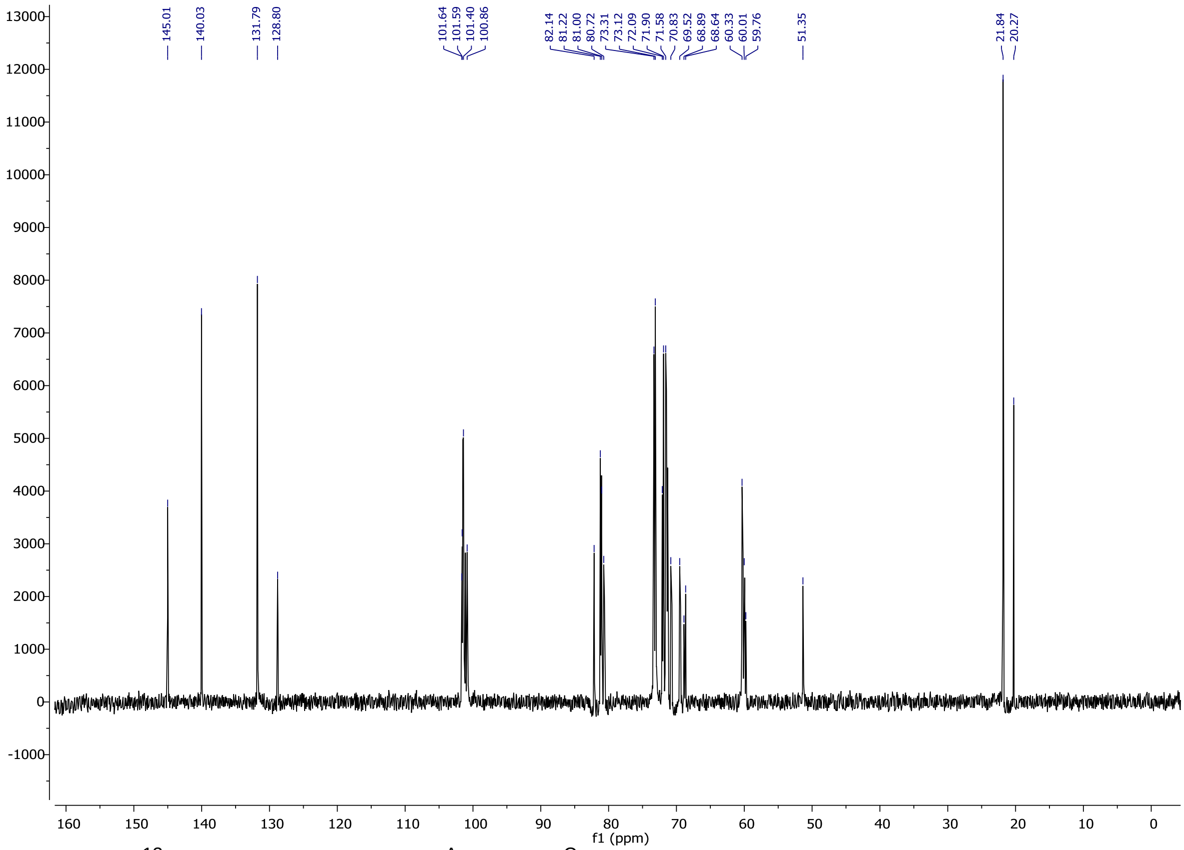

Figure S10: ^{13}C NMR spectrum of 6^{A}-azido-6^{C}-mesitylenesulfonyl-α-CD **8c** (D_2O, 298 K).

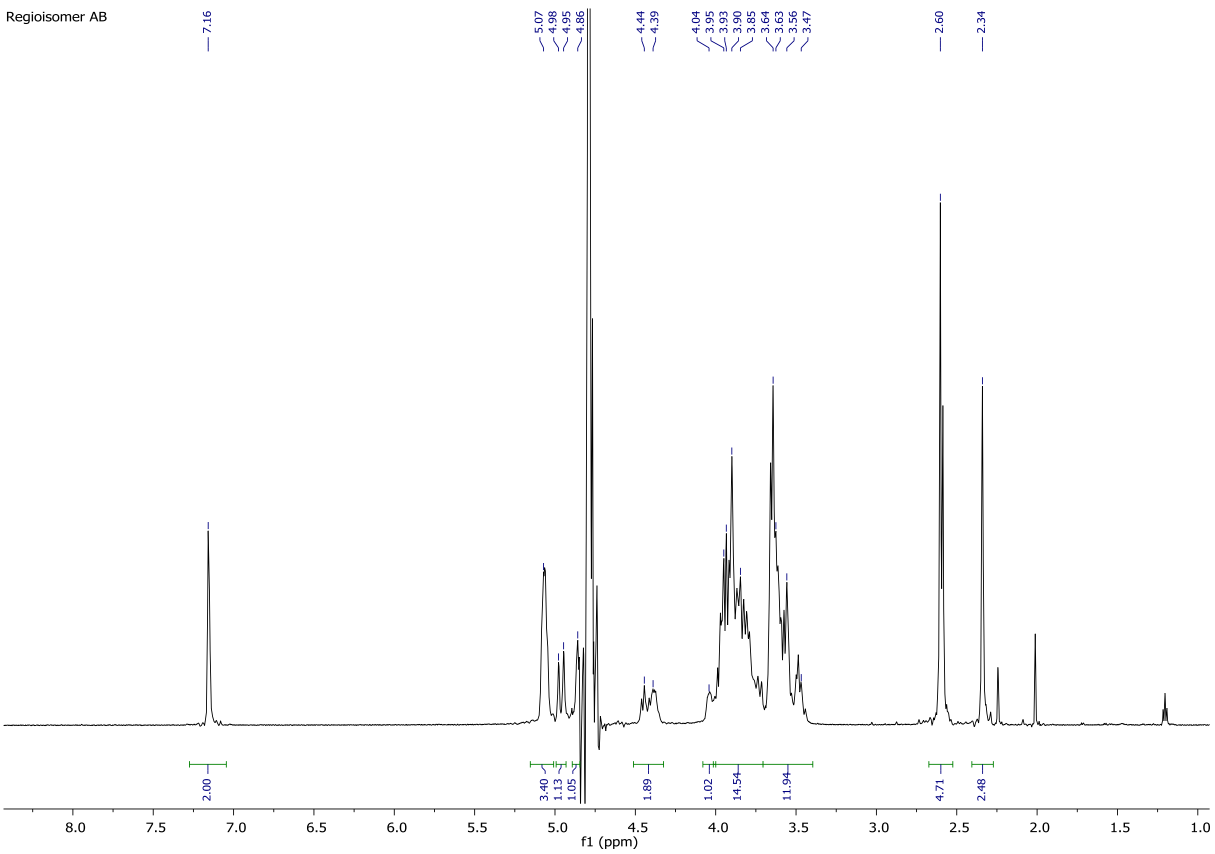

Figure S11: ^{1}H NMR spectrum of 6^{A}-azido-6^{B}-mesitylenesulfonyl-α-CD **8b** (D_2O, 298 K).

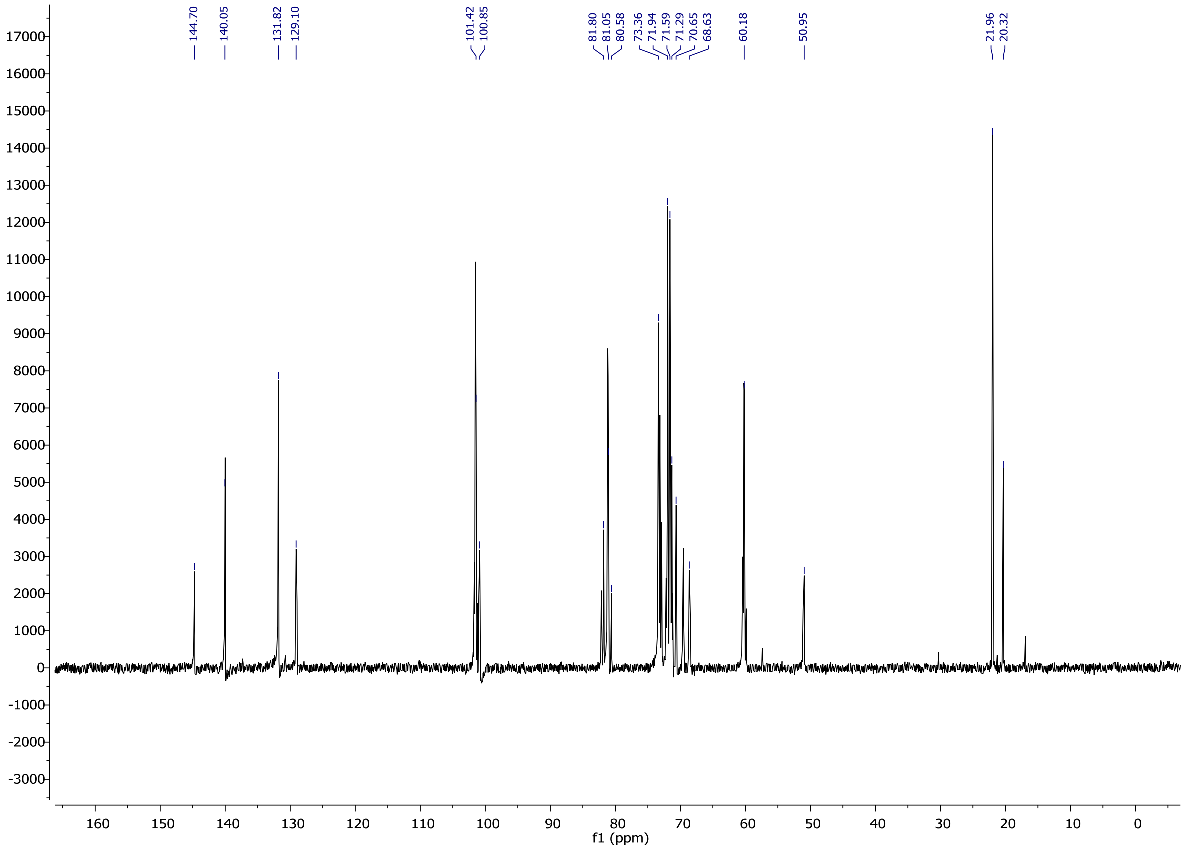

Figure S12: ^{13}C NMR spectrum of 6^{A}-azido-6^{B}-mesitylenesulfonyl-α-CD **8b** (D_2O, 298 K).

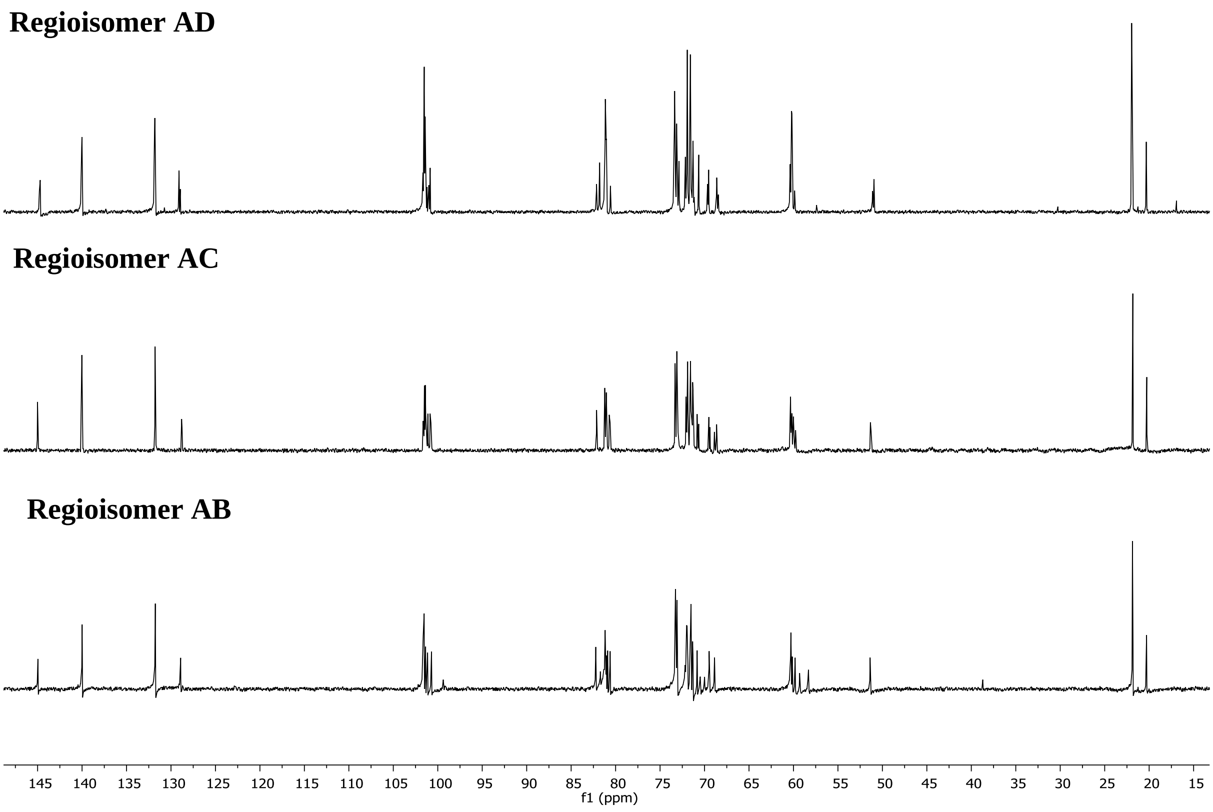

Figure S13: Comparison of ^{13}C NMR spectra of 6^{A}-azido-6^{X}-mesitylenesulfonyl-α-CD regioisomers **8d**, **8c**, **8b** (D_2O, 298 K).

2D NMR spectra of heterobifunctionalized α-CDs

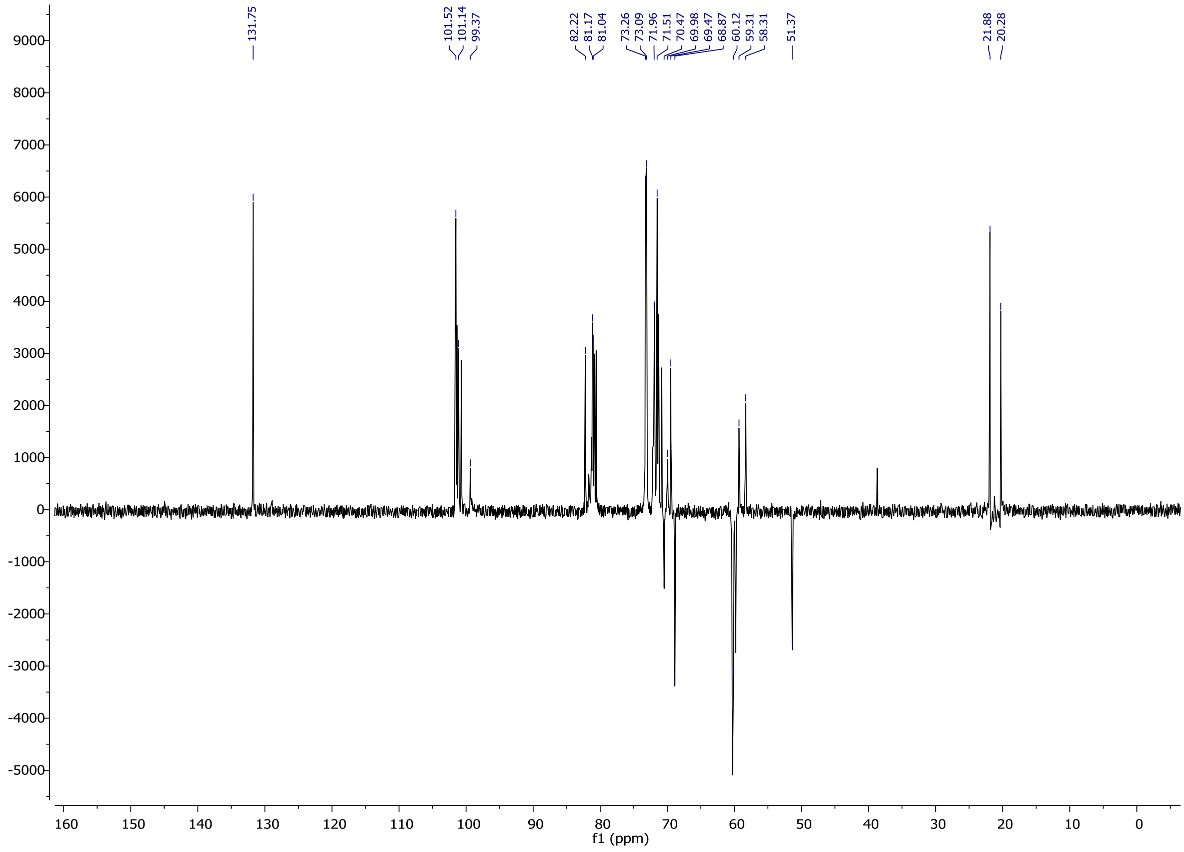

Figure S14: ^{13}C DEPT spectrum of 6^A-azido-6^D-mesitylenesulfonyl-α-CD **8d** (D_2O, 298 K).

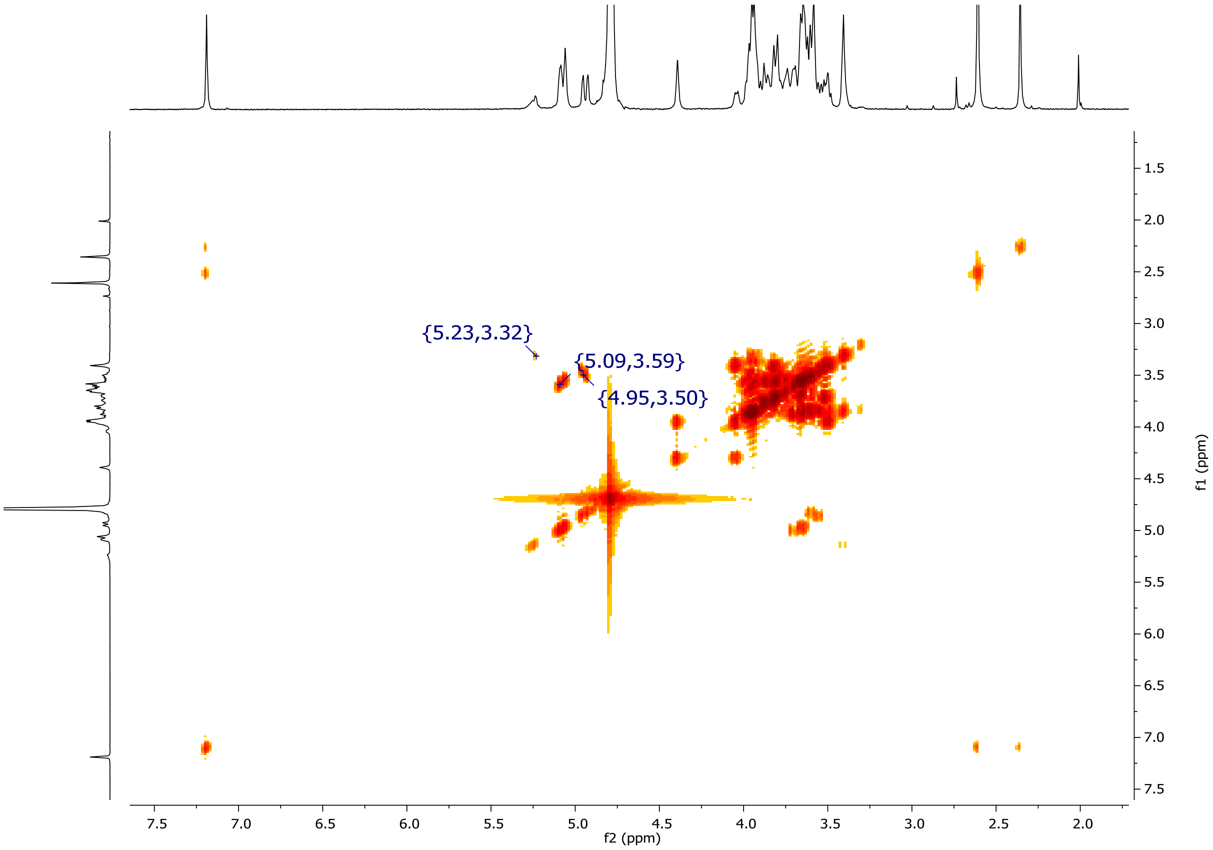

Figure S15: ^1H-^1H COSY spectrum of 6^A-azido-6^D-mesitylenesulfonyl-α-CD **8d** (D_2O, 298 K).

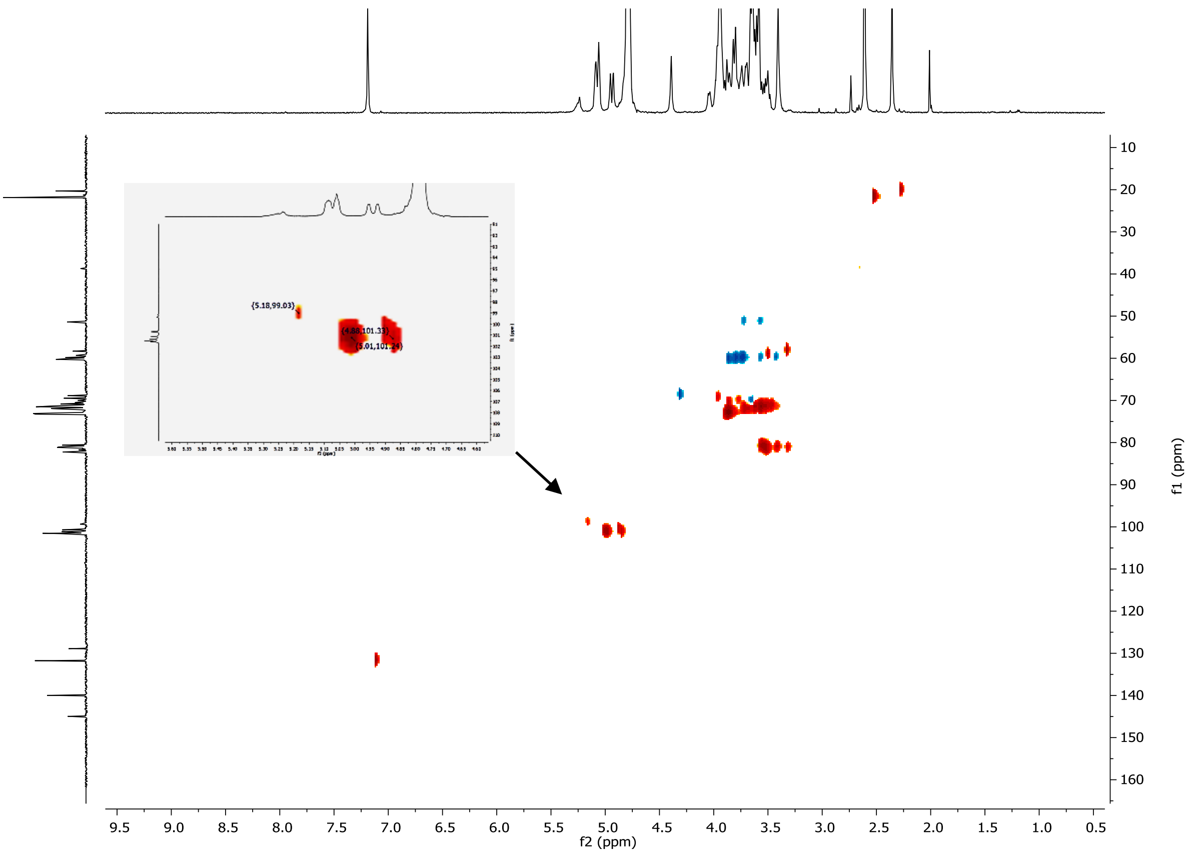

Figure S16: DEPT-edited HSQC spectrum of 6^A-azido-6^D-mesitylenesulfonyl-α-CD **8d** (D_2O, 298 K).

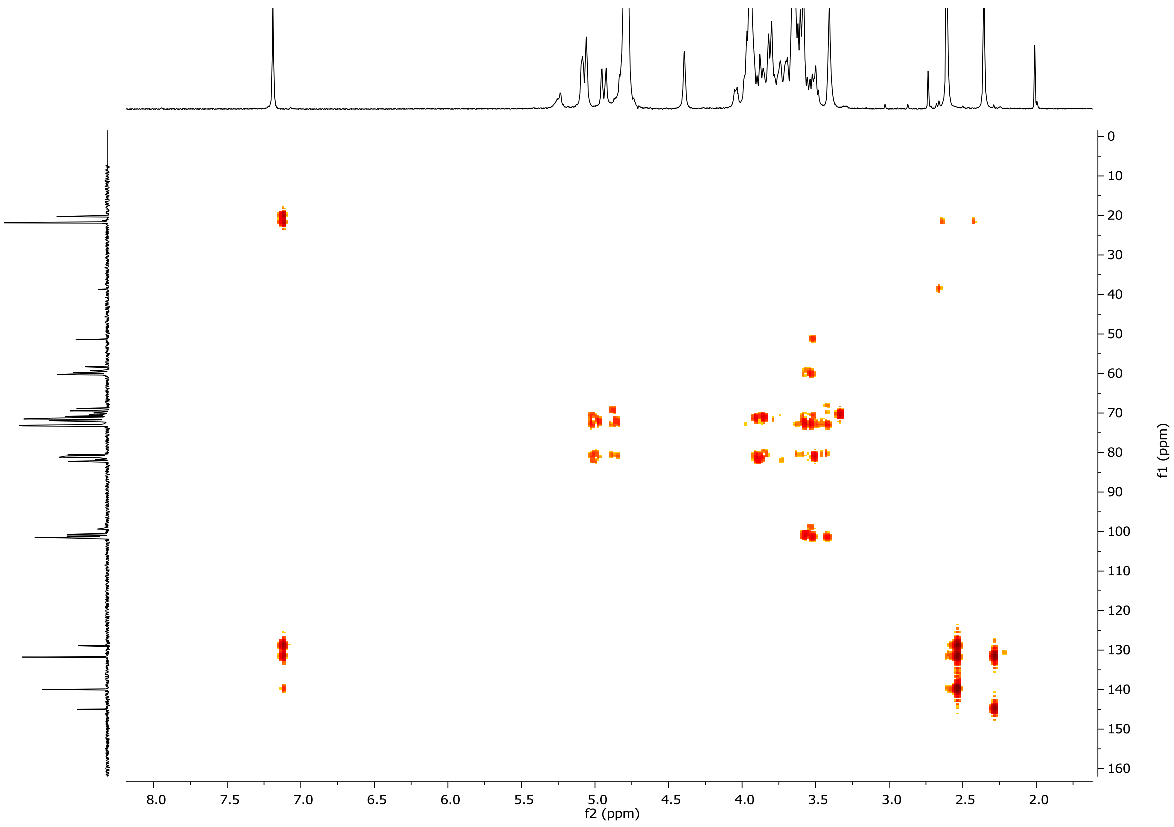

Figure S17: HMBC spectrum of 6^A-azido-6^D-mesitylenesulfonyl-α-CD **8d** (D_2O, 298 K).

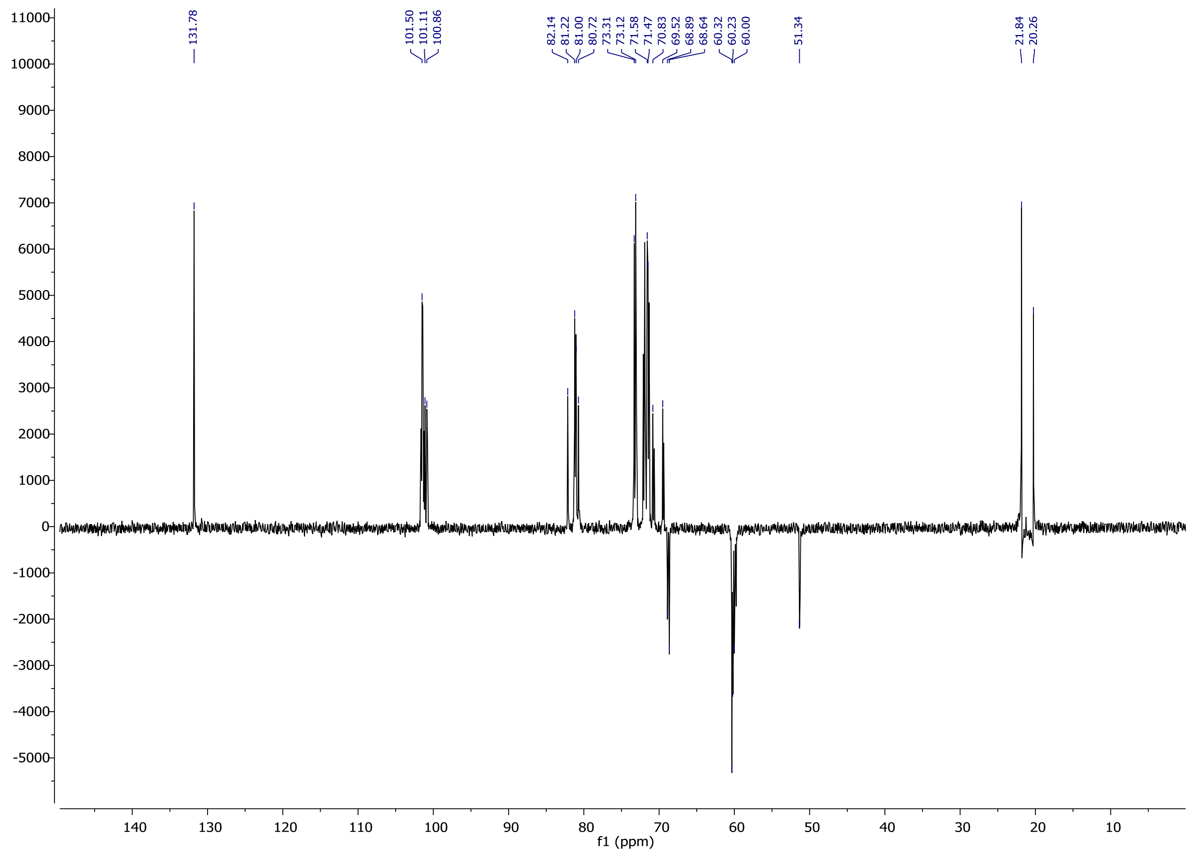

Figure S18: ^{13}C DEPT spectrum of 6^{A}-azido-6^{C}-mesitylenesulfonyl-α-CD **8c** (D_2O, 298 K).

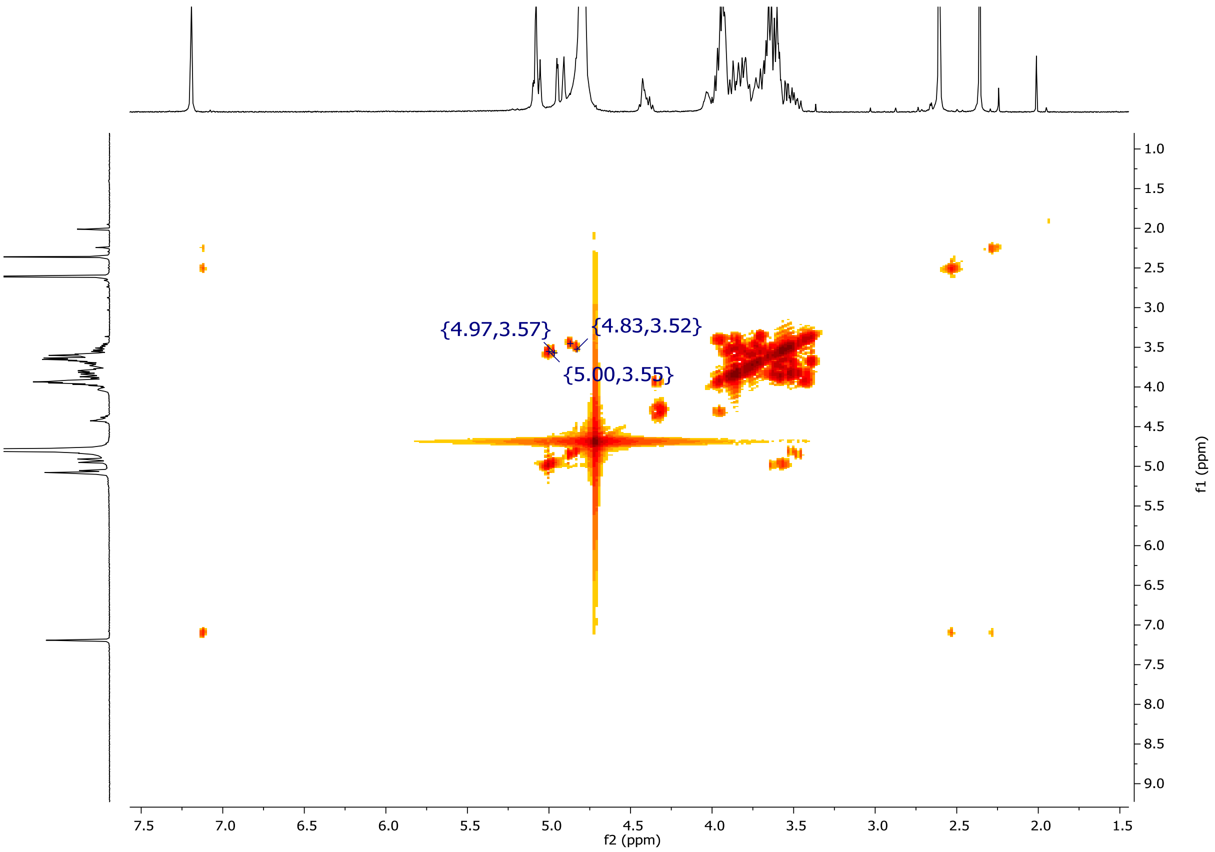

Figure S19: ^{1}H-^{1}H COSY spectrum of 6^{A}-azido-6^{C}-mesitylenesulfonyl-α-CD **8c** (D_2O, 298 K).

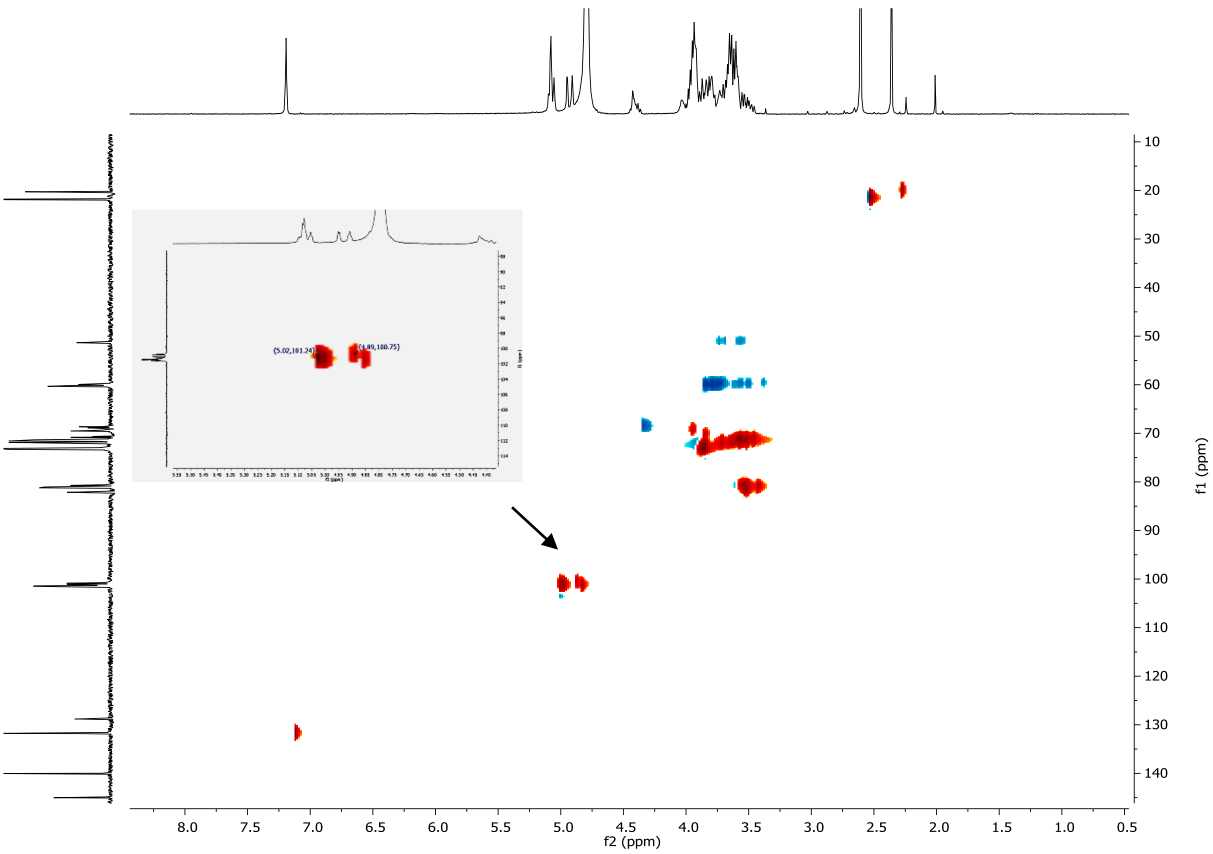

Figure S20: DEPT-edited HSQC spectrum of 6^A-azido-6^C-mesitylenesulfonyl-α-CD **8c** (D_2O, 298 K).

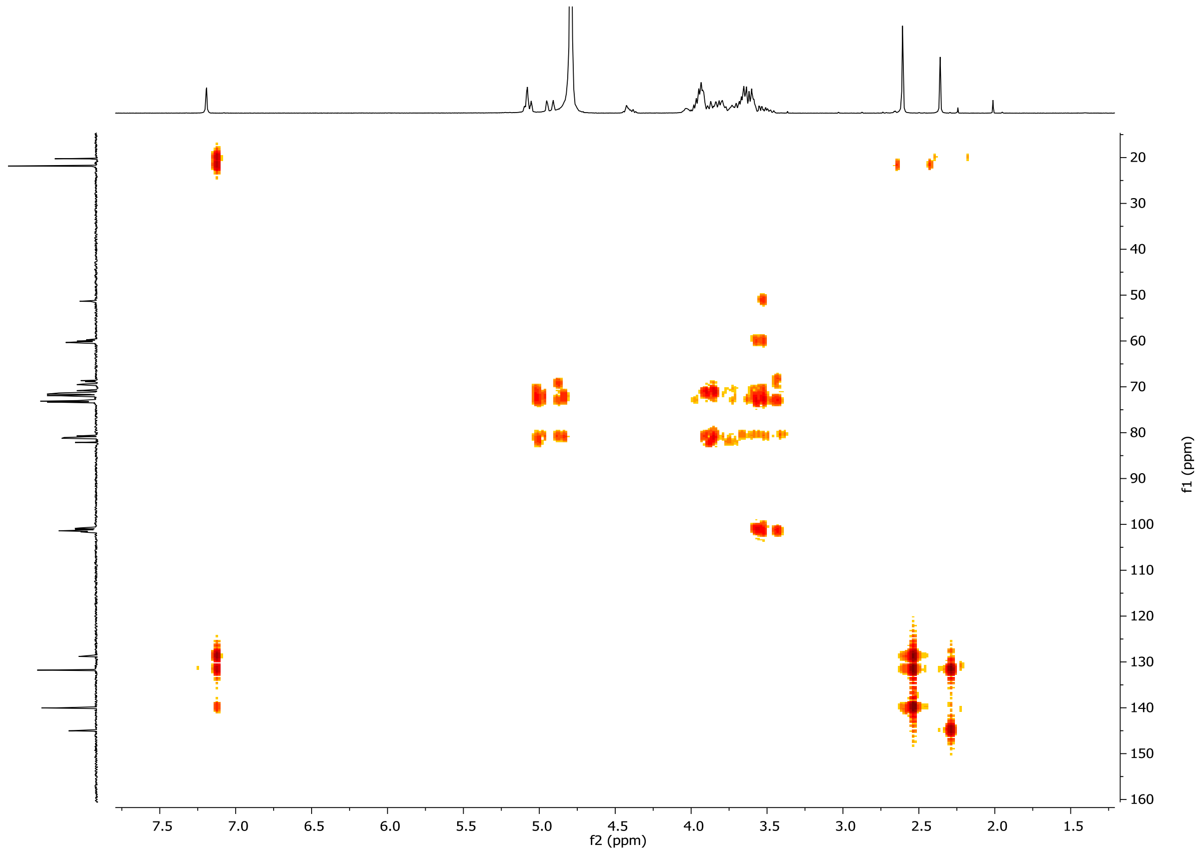

Figure S21: HMBC spectrum of 6^A-azido-6^C-mesitylenesulfonyl-α-CD **8c** (D_2O, 298 K).

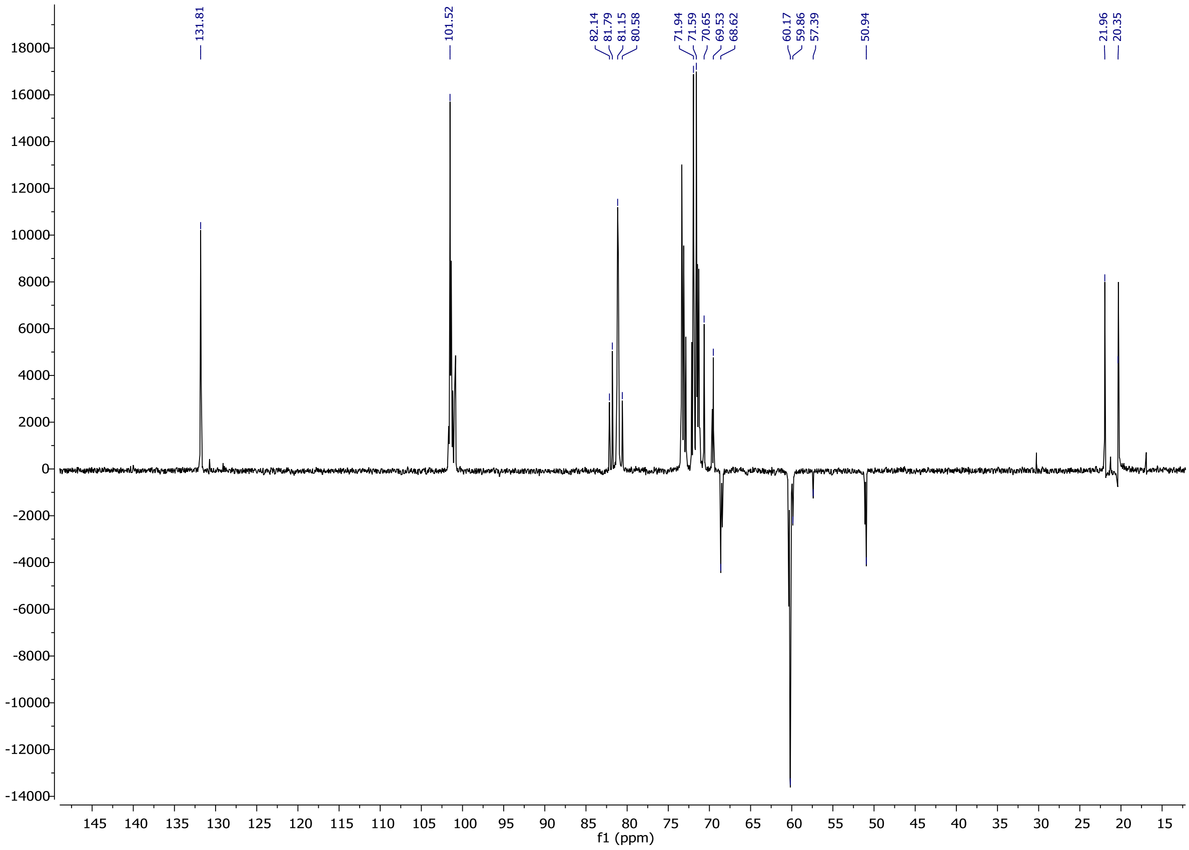

Figure S22: ^{13}C DEPT spectrum of 6^{A}-azido-6^{B}-mesitylenesulfonyl-α-CD **8b** (D_2O, 298 K).

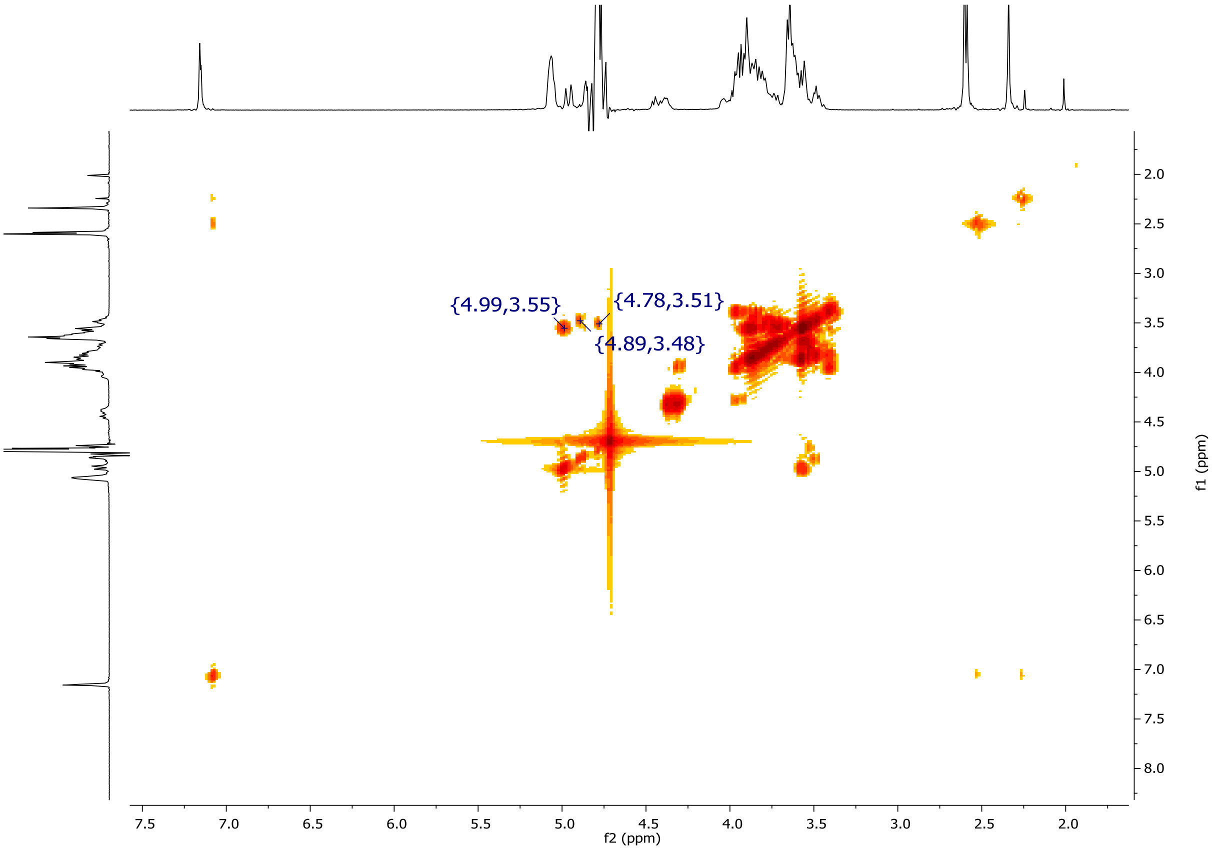

Figure S23: ^{1}H-^{1}H COSY spectrum of 6^{A}-azido-6^{B}-mesitylenesulfonyl-α-CD **8b** (D_2O, 298 K).

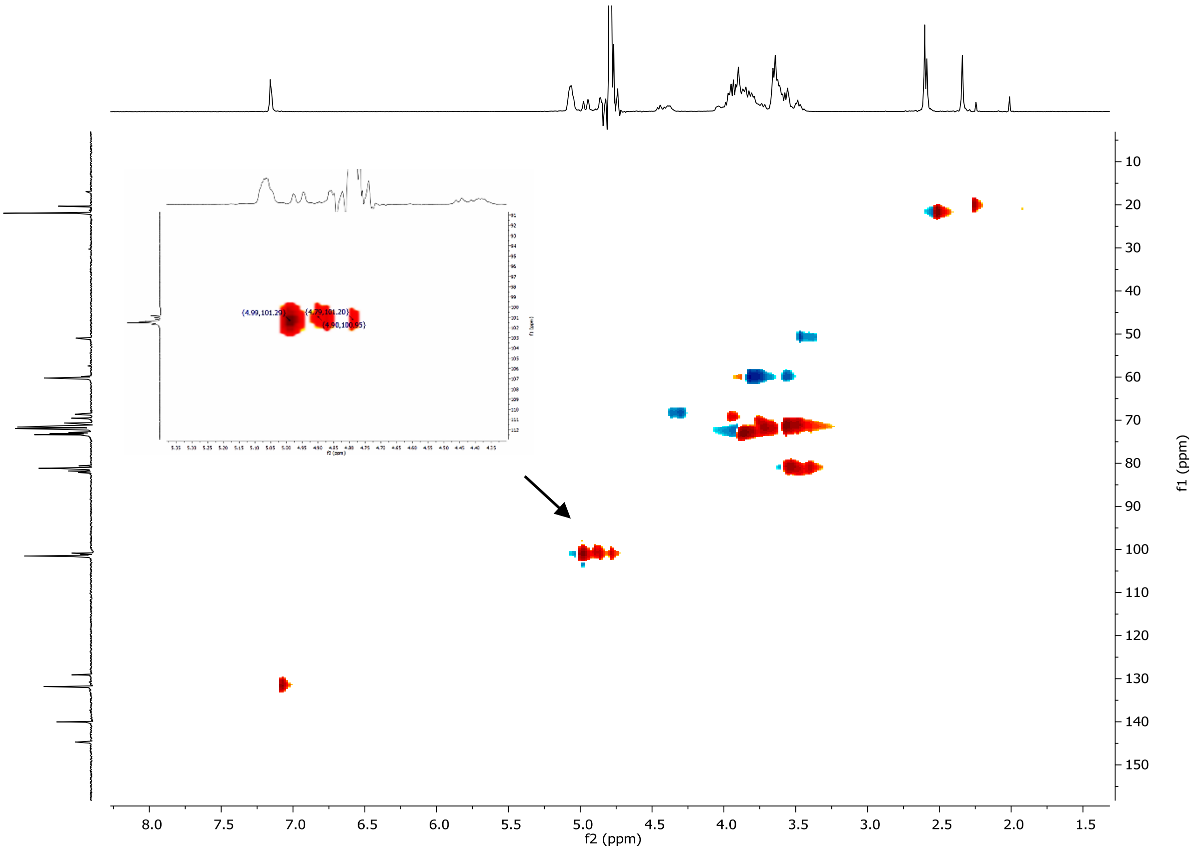

Figure S24: DEPT-edited HSQC spectrum of 6^{A}-azido-6^{B}-mesitylenesulfonyl-α-CD **8b** (D_2O, 298 K).

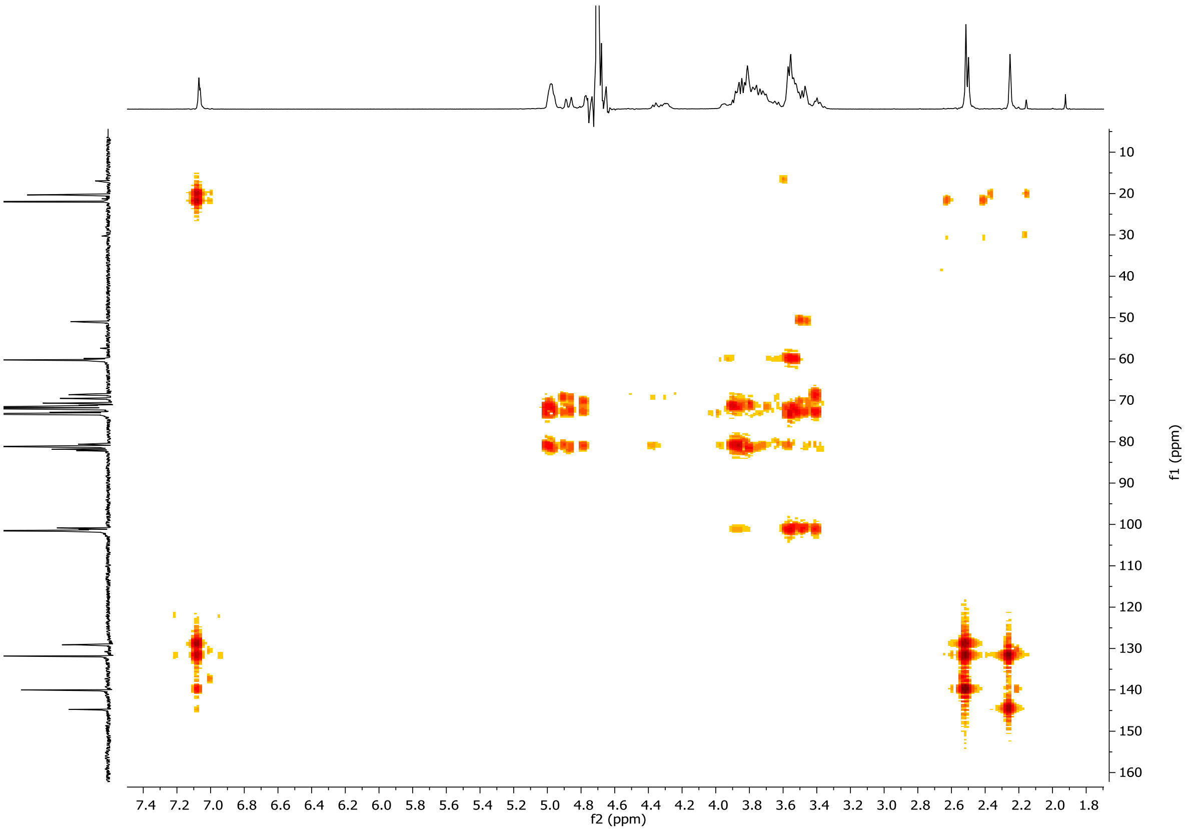

Figure S25: HMBC spectrum of 6^{A}-azido-6^{B}-mesitylenesulfonyl-α-CD **8b** (D_2O, 298 K).

HRMS spectra of prepared compounds

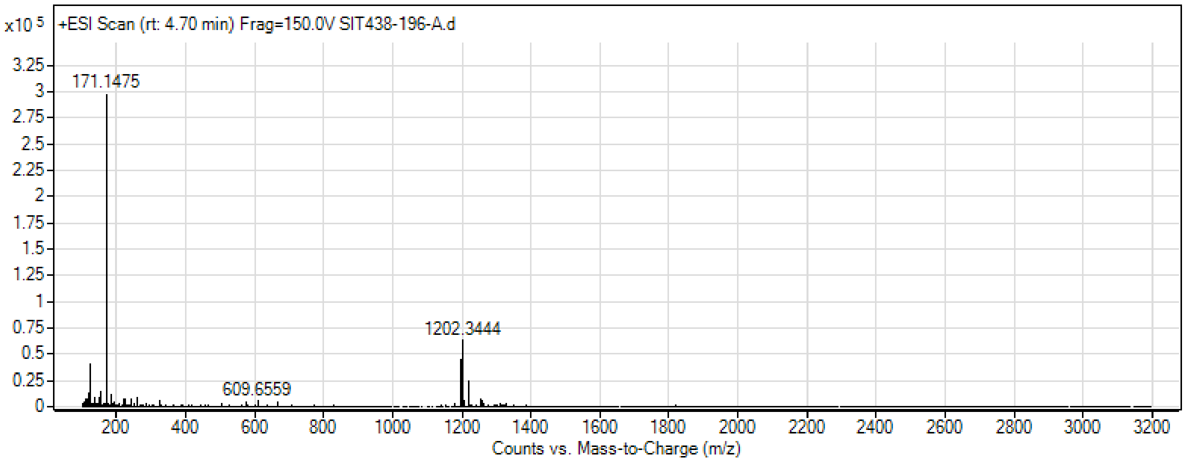

Figure S26: HRMS spectra of 6A-azido-6D-mesitylenesulfonyl-α-CD **8d**.

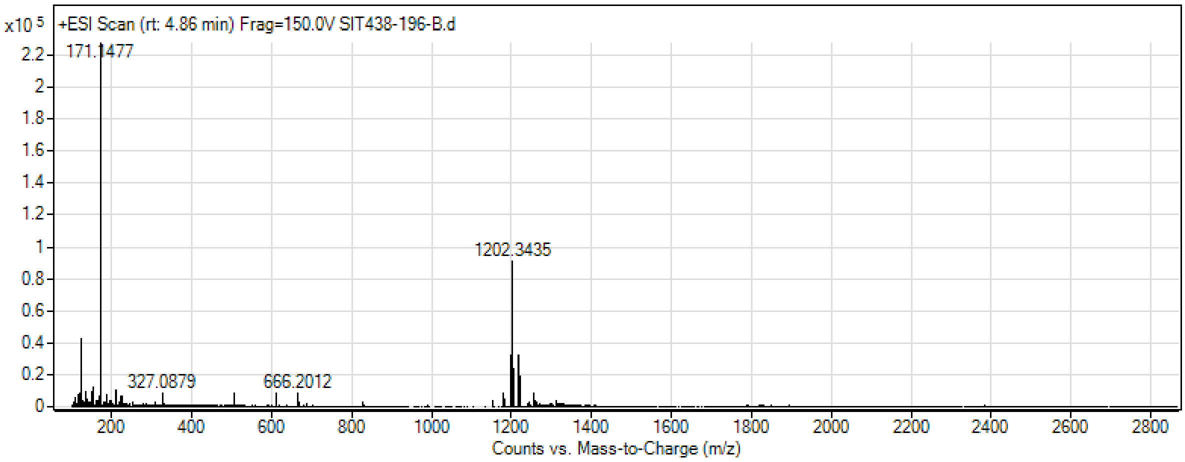

Figure S27: HRMS spectra of 6A-azido-6C-mesitylenesulfonyl-α-CD **8c**.

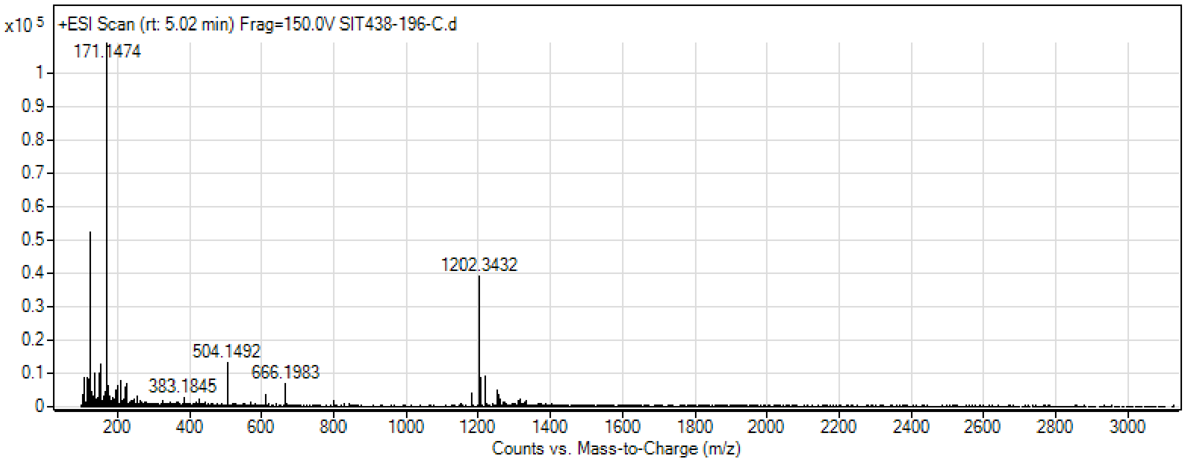

Figure S28: HRMS spectra of 6A-azido-6B-mesitylenesulfonyl-α-CD **8b**.

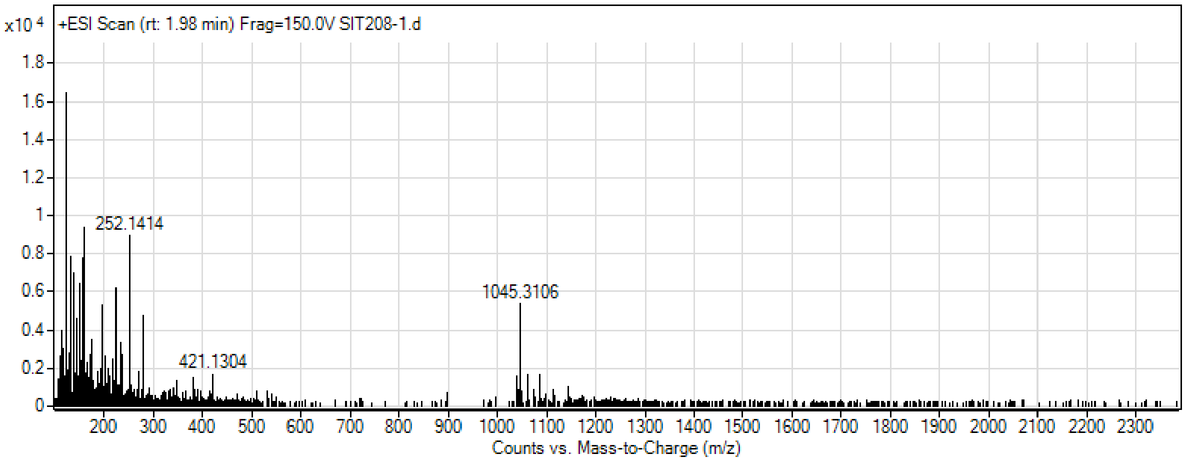

Figure S29: HRMS spectra of 6^A, 6^X-diazido-α-CD **4** (mixture of regioisomers, Reaction 3).
